# Skin fibroblast metabolomic profiling reveals that lipid dysfunction predicts the severity of Friedreich’s ataxia

**DOI:** 10.1016/j.jlr.2022.100255

**Published:** 2022-07-16

**Authors:** Dezhen Wang, Elaine S. Ho, M. Grazia Cotticelli, Peining Xu, Jill S. Napierala, Lauren A. Hauser, Marek Napierala, Blanca E. Himes, Robert B. Wilson, David R. Lynch, Clementina Mesaros

**Affiliations:** 1 Center for Excellence in Environmental Toxicology, Department of Systems Pharmacology and Translational Therapeutics, University of Pennsylvania, Philadelphia, Pennsylvania, USA; 2 Department of Pathology and Laboratory Medicine, Children's Hospital of Philadelphia, Philadelphia, Pennsylvania, USA; 3 University of Alabama at Birmingham, Department of Biochemistry and Molecular Genetics, UAB Stem Cell Institute, Birmingham, Alabama, USA; 4 Department of Neurology and Pediatrics, Children’s Hospital of Philadelphia, Abramson Research, Philadelphia, Pennsylvania, USA; 5 Department of Biostatistics, Epidemiology and Informatics, University of Pennsylvania, Philadelphia, Pennsylvania, USA

**Keywords:** frataxin, ceramides, fatty acids oxidation, triglycerides, phospholipids, lipidomics, lipid remodeling, neurodegenerative disorders, triplet repeat expansion, stable isotope tracing, AcCa, acylcarnitine, ACN, acetonitrile, BHB, β-hydroxybutyrate, Cer, ceramide, CerS, ceramide synthase, DIPEA, N,N-diisopropylethylamine, FRDA, Friedreich’s ataxia, HFIP, 1,1,1,3,3,3-hexafluoro-2-propanol, LC-HRMS, liquid chromatography-high resolution mass spectrometry, PC, phosphatidylcholine, PCA, principal component analysis, PE, phosphatidylethanolamine, PG, phosphatidylglycerol, SCM, significantly changed metabolite, SFA, saturated fatty acid, TG, triglyceride

## Abstract

Friedreich’s ataxia (FRDA) is an autosomal recessive neurodegenerative disorder caused by a triplet guanine-adenine-adenine (GAA) repeat expansion in intron 1 of the FXN gene, which leads to decreased levels of the frataxin protein. Frataxin is involved in the formation of iron-sulfur (Fe-S) cluster prosthetic groups for various metabolic enzymes. To provide a better understanding of the metabolic status of patients with FRDA, here we used patient-derived fibroblast cells as a surrogate tissue for metabolic and lipidomic profiling by liquid chromatography-high resolution mass spectrometry. We found elevated HMG-CoA and β-hydroxybutyrate-CoA levels, implying dysregulated fatty acid oxidation, which was further demonstrated by elevated acyl-carnitine levels. Lipidomic profiling identified dysregulated levels of several lipid classes in FRDA fibroblast cells when compared with non-FRDA fibroblast cells. For example, levels of several ceramides were significantly increased in FRDA fibroblast cells; these results positively correlated with the GAA repeat length and negatively correlated with the frataxin protein levels. Furthermore, stable isotope tracing experiments indicated increased ceramide synthesis, especially for long-chain fatty acid-ceramides, in FRDA fibroblast cells compared with ceramide synthesis in healthy control fibroblast cells. In addition, PUFA-containing triglycerides and phosphatidylglycerols were enriched in FRDA fibroblast cells and negatively correlated with frataxin levels, suggesting lipid remodeling as a result of FXN deficiency. Altogether, we demonstrate patient-derived fibroblast cells exhibited dysregulated metabolic capabilities, and their lipid dysfunction predicted the severity of FRDA, making them a useful surrogate to study the metabolic status in FRDA.

Friedreich’s ataxia (FRDA) is an autosomal recessive neurodegenerative disorder with an incidence of 1 in 29,000 (1). Currently it has no approved treatment (1). The main clinical features in FRDA include gait and limb ataxia, dysarthria, sensory loss, and cardiomyopathy (2). Heart failure from cardiomyopathy is the primary cause of death in the majority of patients with FRDA (3). FRDA is caused by a triplet guanine-adenine-adenine (GAA) repeat expansion in intron 1 of the *FXN* gene that leads to gene silencing and decreased levels of the mitochondrial protein frataxin (4). The number of GAA repeats inversely correlates with frataxin protein level and age of disease onset, both of which determine disease severity (5, 6). The tissues most affected are the heart, dorsal root ganglia, posterior columns of the spinal cord, dentate nucleus, and corticospinal tracts. The exact mechanism by which frataxin deficiency leads to neuro- and cardiodegeneration is not completely understood.

One function of frataxin is in the formation of the iron-sulfur (Fe-S) cluster prosthetic groups that are critical for enzymes in the Krebs cycle (aconitase), oxidative phosphorylation (electron transport chain components of complexes I–III), and fatty acid breakdown (β-oxidation) (7, 8). Frataxin localization in the mitochondria (9) further suggests that mitochondrial dysfunction plays a role in FRDA. Decreased conversion of labeled glucose to acetyl-CoA in platelets from patients with FRDA (10) is consistent with studies that show diminished pyruvate oxidation in FRDA (10). Increased incorporation of labeled palmitate into HMG-CoA, an important intermediate in ketogenesis and sterol synthesis, in patients with FRDA suggests increased fatty acid metabolism through β-oxidation (11). Increased β-oxidation produces FADH_2_ and NADH that can be utilized to maintain the electrochemical gradient across the inner mitochondrial membrane needed for ATP synthesis. Therefore, increased lipid metabolism observed in FRDA could be important to maintain cellular homeostasis during mitochondrial dysfunction.

A recent study found reactive oxygen species-independent accumulation of iron in the nervous system of an FRDA fly model with a mutant frataxin homolog, associated with enhanced sphingolipid synthesis (12). Sphingolipids are linked to increased inflammation (13) and activate 3-phosphoinositide dependent protein kinase-1 (Pdk1) and myocyte enhancer factor-2 (Mef2) to trigger neurodegeneration (12). The findings in the fly model were replicated in a frataxin knockdown mouse model suggesting that the mechanism is evolutionarily conserved (14). PDK1 activity and sphingolipid levels were also elevated in heart tissues of patients with FRDA compared with healthy controls suggesting that a similar pathway may be activated in humans with FRDA (14).

Ceramides are central intermediates in sphingolipid metabolism and have been implicated in several cellular processes including apoptosis (15). Dysregulated ceramides have been the focus of study in a variety of cardiac diseases. High ceramide ratios of Cer 16:0 and 18:0 to Cer 24:0 in plasma are strongly associated with increased risk for major adverse cardiac events (16). Furthermore, increased ceramide levels have been associated with diabetic cardiomyopathy (17) and increased de novo ceramide synthesis has been linked to advanced heart failure (18). The observation of elevated ceramides in FRDA heart tissue raises the question of whether sphingolipids will be dysregulated in other affected and nonaffected tissues.

Ideally, metabolic and lipidomic abnormalities should be studied in the most affected tissues, but frataxin deficiency is present in all tissues to different extents (19). Since it is difficult to sample human cardiac tissue from living individuals, peripheral tissues, such as fibroblasts, can be used as models to study metabolic profiles of FRDA. Fibroblasts in culture have the additional advantage of not being influenced by diet or environment, thus providing a stable system for comparing metabolic flux between patients and controls. Recently, RNA sequencing and gene ontology analysis was used to identify differentially expressed genes between FRDA and healthy control fibroblasts and indicated that fibroblasts are an accessible system to study dysregulated pathways in FRDA (20). In the present study, we used highly sensitive and specific liquid chromatography-high resolution mass spectrometry (LC-HRMS) assays to perform metabolomic and lipidomic profiles in fibroblast cells from patients with FRDA with different disease severities. This study complements the RNA sequencing data and gives new insights into the disease mechanism.

## Materials and methods

### Participants

Nine patients with FRDA and nine healthy controls were entered into the study with no significant differences between the two groups in terms of mean ± SD sampling ages (25.3 ± 5.6 years for FRDA and 27.4 ± 15.5 years for healthy controls) (supplemental Table S1). There were also no significant differences between genders (supplemental Table S1). Punch skin biopsy samples were obtained from participants with approval from the Children’s Hospital of Philadelphia (CHOP) and University of Alabama (UAB) Institutional Review Boards (CHOP IRB #10-007864; UAB IRB #N131204003) (20).

### Chemicals and reagents

DMEM low glucose was purchased from Life Technologies (Carlsbad, CA). HyClone fetal bovine serum (FBS), 1,1,1,3,3,3-hexafluoro-2-propanol (HFIP), isopropanol, and Optima-grade water, methanol (MeOH), methyl-tert-butyl ether and acetonitrile (ACN) were purchased from Thermo Fisher Scientific (Waltham, MA). As detailed in supplemental Table S2, various metabolic internal standards (ISTD) were purchased from Sigma-Aldrich (St. Louis, MO), Cambridge Isotope Laboratories, Inc. (Tewksbury, MA), Toronto Research Chemicals, Inc. (North York, Canada), Cayman Chemicals (Ann Arbor, MI), and Avanti Polar Lipids, Inc. (Alabaster, AL). N,N-Diisopropylethylamine (DIPEA), 5-sulfosalicylic acid, formic acid, and sodium chloride (NaCl) were purchased from Sigma-Aldrich (St. Louis, MO).

### Human fibroblast isolation and culture

Primary fibroblasts from the skin biopsy samples were isolated and established according to detailed procedures as previously published (20, 21). The following cell lines were obtained from the NIGMS Human Genetic Cell Repository at the Coriell Institute for Medical Research: GM04078, GM07522, GM03956, GM02036, GM02671, GM07492, GM03348, GM02153, GM03652, GM02169. Fibroblast cultures were maintained in media consisting of DMEM low glucose and 10% FBS (HyClone). Each line was grown in four technical replicates. Cells were harvested for metabolomic and lipidomic extraction when they reached approximately 80% confluence or 1xE^6^ cells, after incubation with fresh media for 12 h, so all cells would be at the same feeding stage. One of the plates was used for cell counting and all the numbers were within 10% of 1∗E^6^ per plate. For stable isotope tracing experiment, overnight seeded fibroblasts had their media change with fresh medium containing 100 μM ^13^C_16_-palmitate for 6 h. ^12^C-palmitate medium, 100 μM, was used as control for background subtraction. The medium was aspirated, and cells were washed two times with 5 ml 0.9% NaCl before freezing at −80°C.

### Metabolomic extraction

Metabolomic extraction protocol for fibroblasts was adapted from Guo *et al.* (22). A volume of 1 ml of cold 80% MeOH (−80°C), 40 μl of Metabolomics ISTD mix were added to each plate (see concentration for individual metabolites in supplemental Table S2). Cells were scraped and transferred to microcentrifuge tubes in ice. Samples were pulse-sonicated in ice with a sonic dismembranator (Fisher Scientific, Waltham, MA) for 30 s, incubated on ice for 10 min, and then pulse-sonicated again for 30 s. Samples were pelleted by centrifugation at 6,000 *g* for 5 min at room temperature. A volume of 500 μl of supernatant was moved to a clean microcentrifuge tube, dried down under nitrogen, and resuspended in 50 μl of 5% (w/v) 5-sulfosalicylic acid in water. Three microliter injections were used for LC-HRMS analysis with metabolomics method.

### Lipidomic extraction

Lipid extraction protocol for fibroblasts was adapted and modified from previous publication (23). Briefly, 1 ml of cold 80% MeOH from storage at −80°C and 20 μl of Lipidomics ISTD mix were added to each plate (supplemental Table S2). Cells were scraped and transferred to a 10 ml glass Pyrex tube. Buffer, 120 μl, containing 200 mM citric acid and 270 mM disodium hydrogen phosphate (pH 4) was added. Extraction was performed with 2 ml of 1-butanol and 1 ml of water-saturated 1-butanol. The butanol/methanol phase was moved to a clean glass Pyrex tube and evaporated to dryness under nitrogen. The residue was resuspended in 100 μl of methyl-tert-butyl ether/MeOH (1:3 vol ratio). Five microliter injections were used for LC-HRMS analysis with lipidomics method.

### Metabolomics LC-HRMS

Metabolites from the metabolomic extraction were separated using a XSelect HSS C18 column (2.1 mm x 150 mm, 3.5 μm particle size) (Waters, Milford, MA) on a Dionex UltiMate 3000 quaternary UHPLC (Thermo Scientific, Waltham, MA) equipped with a refrigerated autosampler (5^o^C) and column heater (50^o^C). Solvent A was 5 mM DIPEA and 200 mM HFIP and solvent B was 5 mM DIPEA and 200 mM HFIP in methanol. Flow gradient conditions were as follows: 0% B for 2 min at 0.18 ml min^−1^, increased to 1% B for 2 min at 0.2 ml min^−1^, increased to 2% B for 4 min, increased to 14% B for 2 min, increased to 70% B for 2 min, increased to 99% B for 1 min, increased flow rate to 0.3 ml min^−1^ for 0.5 min, increased flow rate to 0.4 ml min^−1^ for 4 min, then washed by decreasing to 0% B for 2.3 min at 0.3 ml min^−1^, decreased to 0.2 ml min^−1^ for 0.2 min, and ending with flow of 0.18 ml min^−1^. Samples were analyzed using a Q Exactive HF (QE-HF) (Thermo Scientific, Waltham, MA) equipped with a heated electrospray ionization source operated in the negative ion mode. Source parameters were the same as described before (22). Data acquisition was performed in negative Full Scan mode 240,000 resolutions. Column effluent was diverted to the QE-HF from 1 to 15 min and then to waste for the remaining time of the run.

### Lipidomics LC-HRMS

Lipids were separated using an Accucore C18 HPLC column (2.1 mm × 100 mm, 2.6 μm particle size) (Thermo Scientific, Waltham, MA) on an UltiMate 3000 quaternary UHPLC equipped with a refrigerated autosampler (10^°^C), and the column heater was set to 35°C. Solvent A was 1/1 ACN/water with 10 mM ammonium formate and 0.1% formic acid. Solvent B was of 10/88/2 ACN/isopropanol/water with 2 mM ammonium formate and 0.02% formic acid. Flow rate was 0.4 ml min^−1^. Flow gradient conditions were as follows: 0 min, 90% A; 1 min, 90% A; 4 min, 60% A; 12 min, 25% A; 21 min, 1% A; 24 min, 1% A; 24.1 min, 90% A; 28 min, 90% A. Samples were analyzed using the QE-HF operated in positive ion mode, then in negative ion mode. Column effluent was diverted to the QE-HF from 2 to 23 min and then to waste for the remaining time of the run.

Data acquisition was performed in Full Scan/ddMS2 mode @ 120,000 resolution. The Full Scan settings were as follows: AGC target, 1e6; Maximum IT, 250 ms; scan range, 250–1800 *m/z*. Top 20 MS/MS spectral (dd-MS2) @ 15,000 were generated with AGC target = 1e5, Maximum IT=25 ms, and (N)CE/stepped nce = 25, 30, 35v. For targeted analysis of ceramide isotopomers, data acquisition was performed in Full Scan/DIA mode. The Full Scan settings is the same with the above setting, for DIA setting, an inclusion list with ceramide was included with scheduled scan window 1 min; other settings: resolution 15,000, AGC target=1e5, maximum IT=auto, isolation window = 1.0 *m/z*, loop count=30.

### Data and statistical analysis

For metabolomics data analysis, quantification of chromatographic peaks was performed with Xcalibur 3.0 (Thermo) from full scan data. The approximate nanogram per plate for each compound was calculated by multiplying the area ratio (area of the compound divided by the area of its internal standard or one internal standard with the closest retention time to the compound of interest, supplemental Table S2) by the nanogram amount of internal standard added to each plate. For compounds that did not have a matching internal standard, an internal standard that had a similar retention time or similar properties was used. For untargeted lipidomics data analysis, peak detection, identification, alignment, and quantification were performed with LipidSearch 4.2 (Thermo). Area for each lipid was normalized by the internal standard from its own class (see supplemental Table S2). For lipids from classes without an internal standard, an internal standard with the closest retention time has been used. For ceramides, two different internal standards were used: Cer 12:0 was used for the normalization of long-chain ceramides and Cer 25:0 was used for the very-long-chain ceramides. The negligible endogenous amounts for Cer 12:0 and 25:0 was confirmed in 1 million of control fibroblast cells spiked with only 18:1 SM–D9 (supplemental Fig. S7A). In addition, calibration curves were constructed for two long-chain ceramides and two very-long-chain ceramides to check that the levels of ceramides in the cells were in the linear range, although absolute concentrations determined for compounds without authentic standards (ceramides with different chain lengths) may not be precise, due to possible differences in instrument response for each chain length. For comparative study, where changes in metabolites level rather than absolute concentration, are most important, the normalization of the peak area with an internal standard from the same class provides better results than no normalization. For targeted ceramide isotopomer analysis, peak integration was performed with Skyline 4.2.0 (MacCoss lab) (24). Principal component analysis (PCA), Heatmap, and volcano plot were generated using R 3.6.3 with package ggplot2, FactoMineR, Factoextra, pheatmap, and EnhancedVolcano. Descriptive statistics were calculated, and unless otherwise stated, data were expressed as mean ± SD. Any data points that were outside the range of mean ± 2∗SD were excluded from statistical analysis. Comparison of patients with FRDA and controls were made by a two-tailed unpaired *t* test. Statistical analysis was conducted using GraphPad Prism v9.0 for Windows.

## Results

### FRDA fibroblasts had moderately compromised energy balance and CoA metabolism

**Fig. 1:**
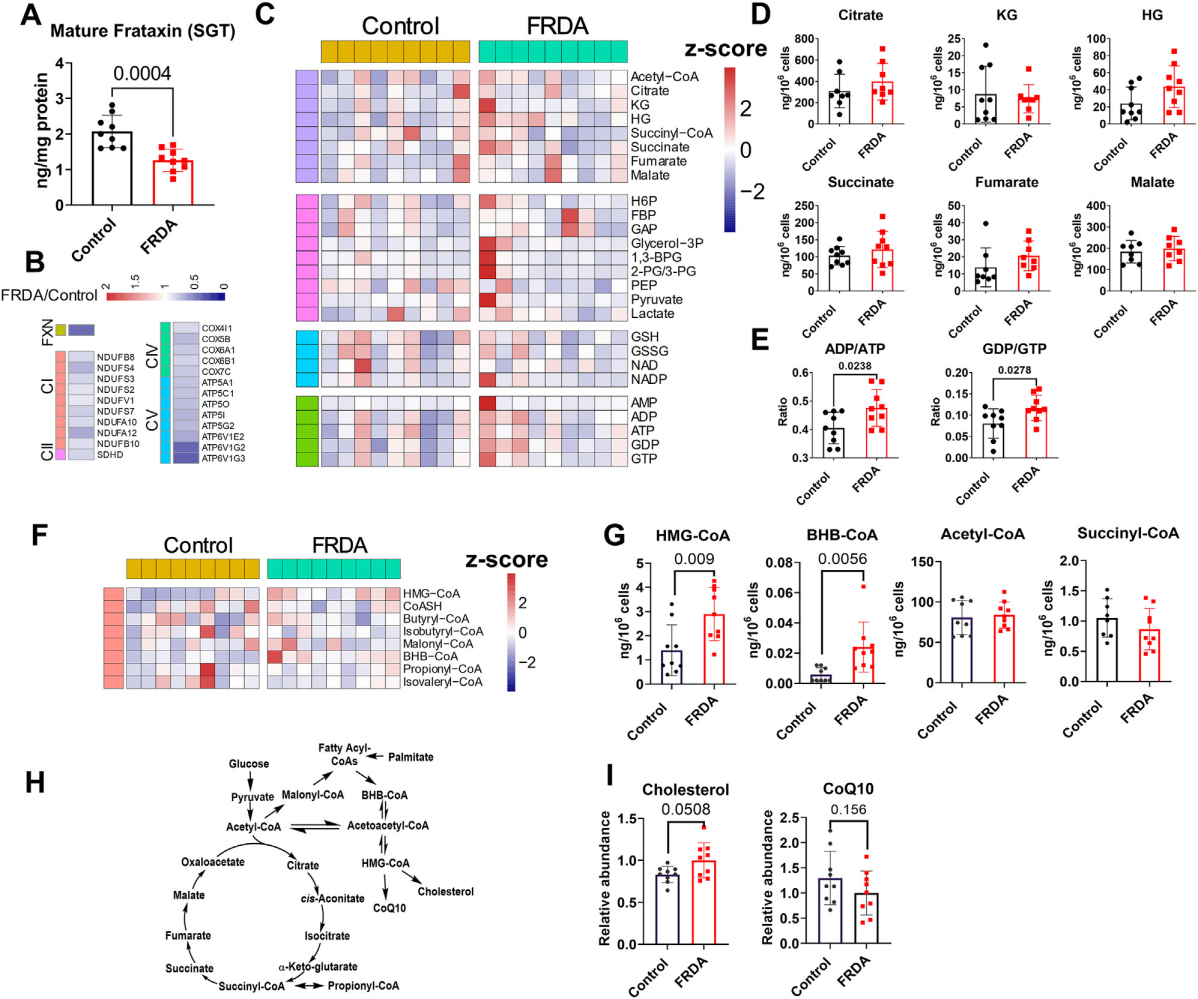
Metabolomic profiling showed compromised energy balance and CoA metabolism in FRDA fibroblast cells (red) compared with healthy controls (black). A: Mature frataxin protein levels determined by liquid chromatography-high resolution mass spectrometry (LC-HRMS). B: mRNA expression of frataxin (FXN) and electron transport chain (ETC) complexes from previous data (26). C: Heatmap shows the major metabolic pathways (from top to bottom: Krebs cycle, glycolysis, redox cycle, and nucleotides). D: The absolute quantification of Krebs cycle intermediates. E: Ratios of ADP/ATP and GDP/GTP. F: Heatmap of acyl-CoAs. G: The absolute abundances of HMG-CoA, BHB-CoA, Acetyl-CoA, and Succinyl-CoA. H: Acly-CoAs-related metabolic pathways. I: The relative abundance for cholesterol and CoQ10. All metabolites were quantified from 1∗E6 cells and the heat maps are presented as the z-scores (mean-centered and divided by standard deviation) of each feature.

Metabolomic profiles from fibroblasts from patients with FRDA and healthy controls (supplemental Table S1) were performed using a previously published method (22) by LC-HRMS after fortification with internal standards from several classes (supplemental Table S2). We examined all the FRDA and control fibroblasts under the microscope (10x zoom); there were no differences in morphology between cells from different lines and passages (supplemental Fig. S1). In order to reduce variability, all cell lines were grown using the same batch of media and FBS and frozen upon reaching 80% confluence. All the cells were later processed at the same time. Four replicates were frozen from each cell line. We quantified the mature form of the frataxin protein from one of the replicates, using a previously published stable isotope dilution LC-HRMS (25) (supplemental Fig. S2). We observed an ∼50% decrease in mature frataxin protein levels in FRDA fibroblasts in comparison with healthy controls (Fig. 1A), which was in agreement with about 44% reduction in FXN mRNA levels from RNA-seq data (26) (Fig. 1B). The transcriptomes of fibroblasts showed significantly decreased expression of genes involved in the electron transport chain and oxidative phosphorylation (26) (Fig. 1B), while the metabolomic profiling showed that the metabolites’ levels in major metabolic pathways, including the Krebs cycle and glycolysis, were not significantly different (Fig. 1C). The absolute levels of Krebs cycle metabolites showed no significant differences between FRDA and control fibroblasts (Fig. 1D). In the glycolysis pathway, only phosphoenolpyruvate was significantly decreased in FRDA patient samples (supplemental Fig. S3). For the patients with FRDA, neither Krebs cycle metabolites nor glycolysis metabolites correlated with the GAA1 repeat expansion (data not shown). The ratios of ADP to ATP and GDP to GTP were significantly increased in FRDA fibroblasts (Fig. 1E), indicating that energy balance was affected by the frataxin deficiency, which is in agreement with a more dramatic decrease of mRNA expressions for complex IV (CIV) genes (Fig. 1B) and decreased ATP5A protein levels (27) in FRDA fibroblasts.

Our metabolomics platform allows for a comprehensive short chain-acyl-Coenzyme A (acyl-CoAs) profiling in the same assay, and the levels of several acyl-CoA molecules, including CoASH, acetyl-CoA, succinyl-CoA, butyryl-CoA, isobutyryl-CoA, malonyl-CoA, HMG-CoA, β-hydroxybutyrate (BHB)-CoA, and propionyl-CoA (Fig. 1F), were quantified. Among these CoAs, BHB-CoA and HMG-CoA were significantly increased in FRDA fibroblasts (Fig. 1G), replicating the observation from the FRDA platelets (11). Figure 1H shows the metabolic pathways starting from glucose or fatty acids that contribute to the production of BHB-CoA and HMG-CoA, pointing to increased fatty acid oxidation in FRDA. We analyzed the downstream metabolites of HMG-CoA (by the lipidomics method, see further), including cholesterol and CoQ10; a slight increase in cholesterol was found in FRDA fibroblasts (Fig. 1I) (*P*=0.051), but no significant difference in CoQ10 level was observed between FRDA and control fibroblasts (Fig. 1I). Levels of acetyl-CoA and succinyl-CoA, two of the most abundant CoA species in mammalian cells, did not differ between FRDA and control fibroblasts, most likely due to other compensatory mechanisms feeding into the CoAs involved in the Krebs cycle (28). In agreement with the similar levels found in FRDA and control fibroblasts, there was no correlation for most of the CoAs levels with GAA repeat length (supplemental Fig. S4B), but BHB-CoA and HMG-CoA had *P* values around 0.07. Untargeted metabolomics data did not identify any other significantly different metabolites between FRDA and control fibroblasts.

### FRDA fibroblasts have a considerably dysregulated lipid profile

**Fig. 2:**
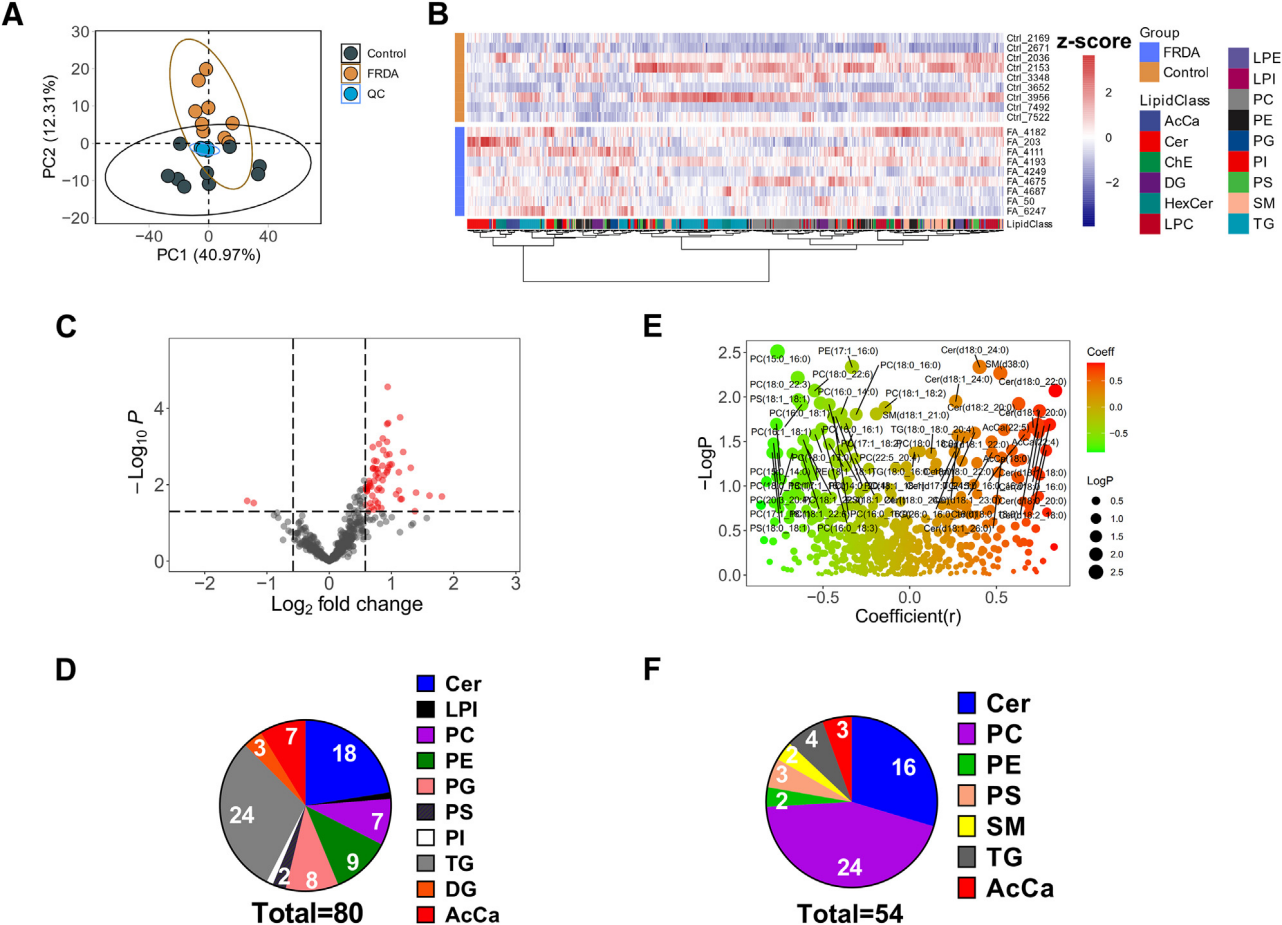
FRDA fibroblast cells had dramatically dysregulated lipid metabolism in comparison with healthy control cells. A: The score plot of principle component analysis (PCA) of lipidomic profiles separates control (black) and FRDA fibroblast cells (yellow). B: Heatmap showed clustering of different classes of lipids between the two groups. The heatmap is presented as the z-scores (mean-centered and divided by standard deviation) of each feature. C: Volcano plot showing the dysregulated lipids filtered by *P* values and fold change. D: Pie chart showing the numbers of isomers from each class of the dysregulated lipids with *P* < 0.05 and fold change >1.5 (from the volcano plot). E: Pearson correlation of the lipid levels from the FRDA fibroblast cells with the GAA1 repeat length. F: Pie chart showing the number of isomers from each lipid class correlated with GAA repeat length with *P* < 0.05 (from the Pearson correlation). All metabolites were quantified from 1∗E6 cells.

Lipidomic profiling was performed on the same FRDA and control fibroblast cells used for metabolomics and frataxin measurements to establish the effect of frataxin deficiency on the lipid metabolism. A reverse-phase LC-HRMS method was employed to analyze more than 500 lipids, including acylcarnitines (AcCas), ceramides (Cers), cholesterol esters, diglycerides, hexosyl-ceramides, lysophosphatidylcholines, lysophosphatidylethanolamines, lysophosphatidylinositols, phosphatidylcholines (PCs), phosphatidylethanolamines (PEs), phosphatidylglycerols (PGs), phosphatidylinositols, phosphatidylserines (PSs), sphingomyelins (SM), and triglycerides (TGs). PCA was used to compare the similarities of the lipidomic profiles between FRDA and control fibroblasts. The score plot of the PCA model showed that the FRDA and control fibroblasts are clearly separated on the PC2 (Fig. 2A), implying frataxin deficiency induced changes in the lipid profile. The quality control samples that were obtained by mixing equal parts from all the lines clustered in a tight area between the two groups (Fig. 2A). The heatmap showed two clearly separated clusters of lipids, generated by the ward.D clustering method and Euclidean clustering distance calculation (Fig. 2B). For this analysis, each lipid was identified using the library data from Lipids Search 4.2 (Thermo) based on the exact mass within 5 ppm accuracy and the presence of at least two positively identified fragments. Areas under the curve for each lipid were normalized to an internal standard (supplemental Table S2) that belonged to the same lipid class to account for extraction efficiency and ion suppression. We generated the *P* value using *t* test and fold changes (FCs) that were defined as the ratio of FRDA to control average response measured by the signal intensity for each ion. Significantly changed metabolites (SCMs) were filtered by *P* value less than 0.05 and FC higher than 1.5 (Fig. 2C), which resulted in 80 lipids (Fig. 2D). Ceramides were the class with a largest number of affected isomers after TGs (Fig. 2D). Several carnitines were identified here too. Given that the FRDA disease severity correlates with increasing number of GAA repeats on the GAA1 allele (29), we performed a Pearson correlation between the GAA1 repeat length and each lipid identified (Fig. 2E). A total of 54 lipids positively or negatively correlated with GAA1 repeat length (Fig. 2F), including 16 ceramides, 24 PCs, 2 PEs, 3 PSs, 2 SMs, 4 TGs, and 3 AcCas. Next, we performed Pearson correlation analysis between mature frataxin protein levels and identified lipids. A total of 49 lipids, including 6 ceramides, showed a good correlation (*P* less than 0.05) (and 20 ceramides with *P* value less than 0.1) (supplemental Fig. S5).

### Ceramide accumulated in FRDA fibroblasts and correlated with GAA repeat length and frataxin levels

**Fig. 3:**
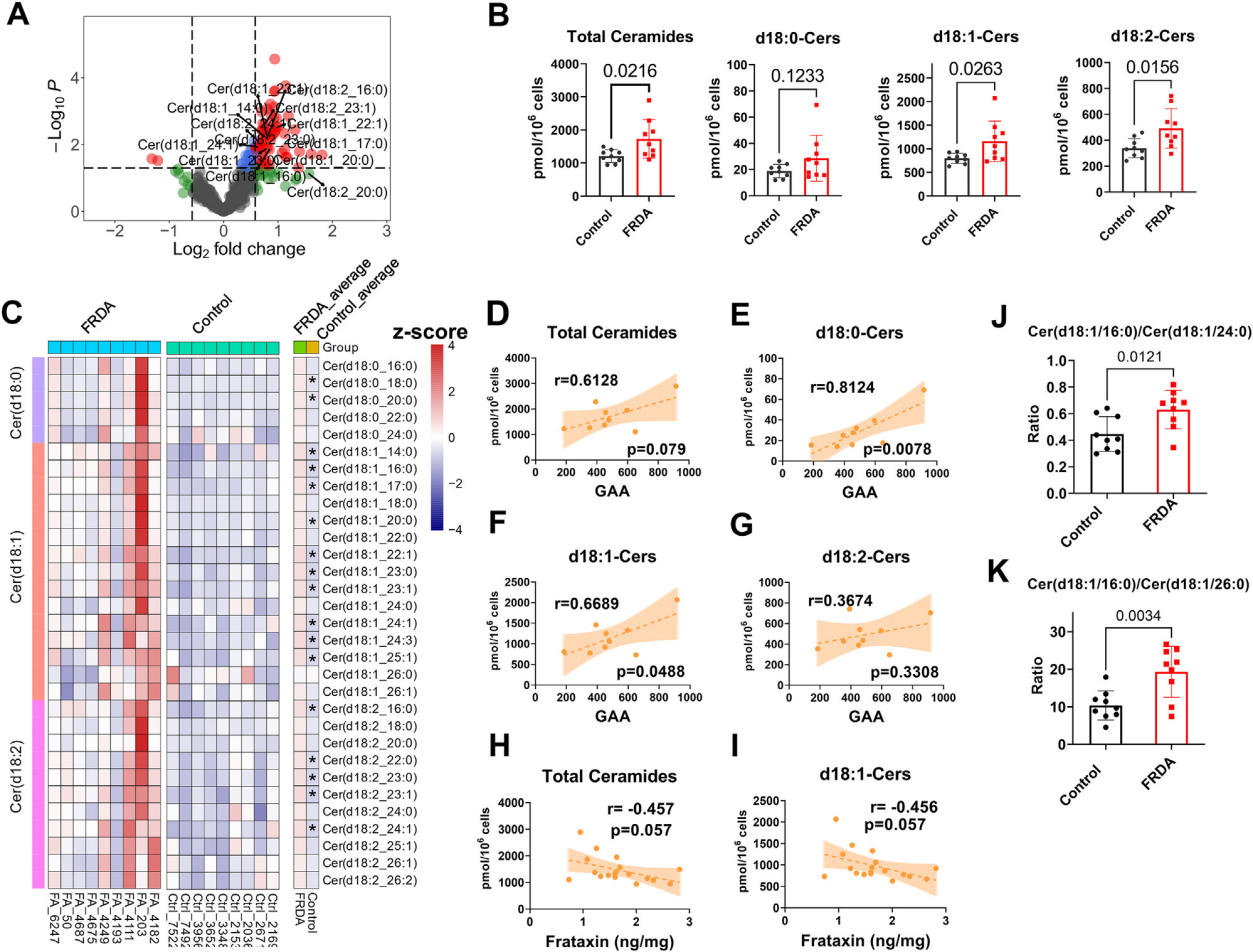
Ceramides accumulated in FRDA fibroblast cells and correlated with known biomarkers of disease, GAA1 repeat length and frataxin levels. A: The volcano plot with labeled ceramides. B: The total levels of ceramides, d18:0-Cers, d18:1-Cers, and d18:2-Cers in control and FRDA fibroblast cells. C: Heatmap showing individual isomers, ∗ indicates *P*<0.05. The heatmap is presented as the z-scores (mean-centered and divided by standard deviation) of each feature. D–G: Pearson correlation between total ceramide levels, d18:0-Cers, d18:1-Cers, and d18:2-Cers, and GAA repeat length. H–I: Pearson correlation between total ceramide and d18:1Cer levels and frataxin levels. J: The ratio of Cer(d18:1/16:0) to Cer(d18:1/24:0). (K) The ratio of Cer(d18:1/16:0) to Cer(d18:1/26:0). All metabolites were quantified from 1∗E6 cells.

Combing SCMs, GAA1 correlated lipids, and frataxin correlated lipids, we found that all had in common a large number of ceramides. Among the 80 SCMs, 18 ceramides were increased in FRDA fibroblasts (Fig. 3A). These ceramides are categorized into dihydroceramides (d18:0-Cers), classical ceramides (d18:1-Cers), and ceramides (d18:2-Cers), corresponding to sphinganine, sphingosine, and sphingadiene as the backbone. The total ceramides were significantly higher in FRDA fibroblasts when compared with the control fibroblasts (*P*=0.028) (Fig. 3B). d18:0-Cers were slightly increased in FRDA, with two long-chain Cers having a *P* value less than 0.05 (Fig. 3C). d18:1-Cers showed an overall increase in FRDA fibroblasts (Fig. 3B), with most of the long-chain Cer having a *P* value less than 0.05 (Fig. 3C). d18:2-Cers are a relatively new class of ceramides (compared with the classical d18:1-Cers) and are supposed to have distinct functions from the d18:1-Cers species (30). d18:2-Cers were also upregulated in FRDA fibroblasts (*P*=0.012) (Fig. 3B). Levels of other sphingolipids, including sphingosine, sphinganine, sphingosine-1-P, Hex1Cer, Hex2Cer, Hex3Cer (supplemental Fig. S6), and SM (data not shown) showed no significant difference between FRDA and control fibroblasts.

It is worth mentioning that we could not identify in the fibroblast cells Cer (18:1/20:4) which was one of the most increased in the heart tissues from patients with FRDA reported by the Bellen group. (14) We did not have access to heart tissue from patients with FRDA, but we tested three individual healthy heart tissues and could not identify Cer (18:1/20:4) (data not shown). Cer(18:1/20:4) does not fit the known acyl-CoA specificity of mammalian CerS1-6, and we would not be surprised if the LC/MS signal has been mixed up with a different compound in the publication of the Bellen group. (14)

The total levels of ceramides slightly correlated with the GAA1 repeat length (Fig. 3D, r=0.6, *P*=0.066). d18:0-Cers (Fig. 3E, r=0.8, *P*=0.014) and d18:1-Cers (Fig. 3F, r=0.7, *P*=0.05) were more strongly correlated with the GAA1 repeat length than d18:2-Cers (Fig. 3G, r=0.4, *P*=0.251). Individual ceramides belonging to d18:0-Cers and d18:1-Cers showed strong positive correlation with GAA repeat length with r > 0.7 and *P* < 0.05 (supplemental Fig. S8). Interestingly, these same ceramides have found to be elevated in heart tissue from patients with FRDA (14). Similarly, we found that total ceramides (Fig. 3H, r=-0.5, *P*=0.054) and d18:1-Cers (Fig. 3I, r=-0.5, *P*=0.053) negatively correlated with the frataxin levels, and most of the classical ceramides (d18:1-Cer) had negative correlations with the frataxin levels with *P*<0.1 or around 0.05 (supplemental Fig. S9). Furthermore, the ratios of long-chain ceramides to very-long-chain ceramides were significantly elevated in FRDA fibroblasts (Fig. 3J, K). The total amount of ceramides and their individual distribution is shown in supplemental Fig. S13 top panel, and the same representation was used for the SM (lower panel, supplemental Fig. S13).

### Transcriptomic data

Supporting the ceramides dysregulation, the analysis of the transcriptomic data (26) identified several transcripts that were highly dysregulated (*P* values < 0.01; false discovery rate <0.050) and could be mapped to the “Sphingolipid metabolism” (KEGG pathway map hsa 00600) (supplemental Fig. S10).

### Stable isotope tracing demonstrated increased ceramide synthesis in FRDA fibroblasts

**Fig. 4:**
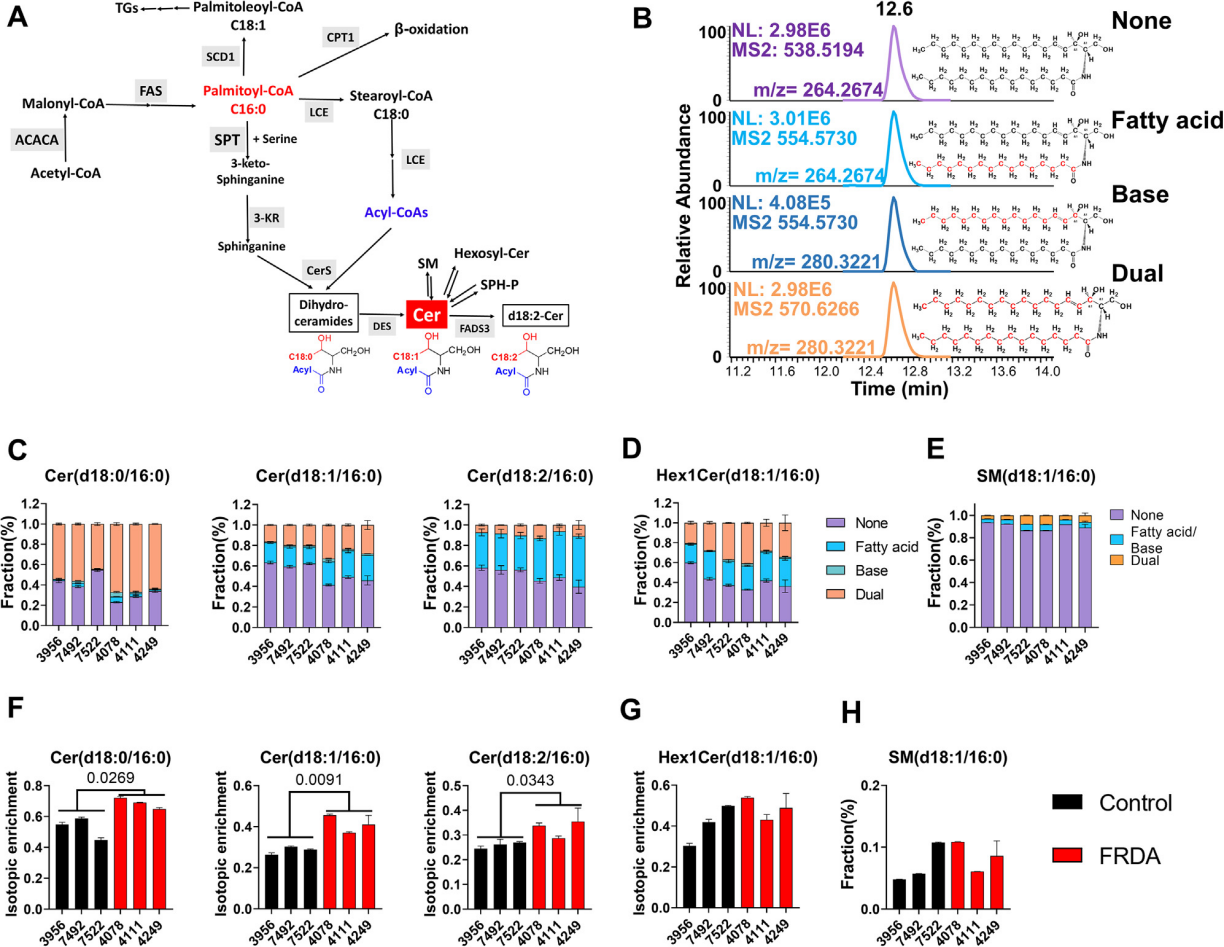
Stable isotope tracing experiments demonstrated higher ceramide synthesis flux in FRDA fibroblast cells. Fibroblast cells from three Control lines (3956, 7492, and 7522) and three FRDA lines (4078, 4111, and 4249) were used for the isotopic tracing experiment. A: Ceramides de novo synthesis relays on acyl-CoAs. B: Chromatograms from parallel reaction monitoring (PRM) helped distinguished between fatty acids labeled (blue) and the sphingoid base labeled (red), to monitor the levels of all ceramide forms from [^13^C_16_]-palmitate. The mass isotopomer distributions of representative (C) ceramides, (D) Hex1Cer, and (E) SM. The isotopic enrichments of representative (F) ceramides, (G) Hex1Cer, and (H) SM.

To investigate if ceramides accumulation in FRDA fibroblasts was due to increased de novo biosynthesis, increased activity in the “salvage pathway,” or hydrolysis of more complex sphingolipids (Fig. 4A), we performed a stable isotope tracing experiment using [^13^C_16_]-palmitate in several fibroblast lines. A targeted-HRMS strategy-parallel reaction monitoring was used for the analysis of mass isotopomer distribution of ceramide or Hex1Cer (SMs were quantified using MS1 precursor for analysis of mass isotopologue distribution). This method allowed us to sensitively and selectively detect ceramide or Hex1Cer with no-chain-labeled, fatty-acid-chain-labeled, sphingosine-base-labeled, and dual-labeled forms simultaneously (Fig. 4B). We used three independent cell lines for FRDA [each with a relatively low GAA repeat length (supplemental Table S1) as the longer GAA repeat cell lines grow slower] and three healthy control cell lines. As expected, ceramides containing the C16:0 chain had the highest labeling fraction from all the ceramides (Figs. 4C and S11A). The FRDA fibroblast cells (lines 4078 and 4111 with GAA1 <500; and line 4249 with GAA1 >500) had higher fractions of labeled forms of Cer(d18:0/16:0), Cer(d18:1/16:0), and Cer(d18:2/16:0) (Fig. 4F, red bars) than control fibroblasts (lines 3956, 7492, 7522) (Fig. 4F, black bars), especially for fatty acid chain labeled and dual labeled forms (Fig. 4B). However, we observed no difference in the labeling fraction of Hex1Cer(d18:1/16:0) and SM(d18:1/16:0) between FRDA and control fibroblasts (Fig. 4D, E). SM(d18:1/16:0) was tentatively identified as it is one of the most abundant SM in fibroblast cells and has been identified based on the same retention time with an authentic standard (supplemental Fig. S12A) (used for testing the liner response for quantification), and the MS2 spectra from negative mode (supplemental Fig. S12E).

Isotopic enrichment was calculated for each ceramide, Hex1Cer, and SM after natural abundance correction according to a previous method (31). FRDA fibroblasts showed higher isotopic enrichments for all the ceramides containing the 16:0 chain, Cer(d18:0/16:0), Cer(d18:1/16:0), and Cer(d18:2/16:0) when compared with control fibroblasts (Fig. 4F). There were no differences in isotopic enrichment of Hex1Cer (d18:1/16:0) or SM (d18:1/16:0) between FRDA and control fibroblasts (Fig. 4G, H).

Cer(d18:0/16:0) and Cer(d18:1/16:0) were more similarly increased in all three tested FRDA lines, but the Cer(d18:2/16:0) was differently increased between cell lines. We also observed higher isotopic enrichment for very-long-chain ceramides, such as Cer(d18:1/24:0) and Cer(d18:1/24:1) (supplemental Fig. S11B), which was consistent with the lipidomic profiling that showed that the long-chain ceramides were more significantly increased than the very-long-chain ceramides (Fig. 3C, J and K). The longer-chain ceramides require additional enzymatic steps for fatty acid elongation, so it is reasonable to assume that longer incubation times will lead to increased labeling for longer-chain ceramides. Combined, these results provide evidence that the FRDA fibroblasts, even though not part of an affected tissue, accumulated ceramides in comparison with healthy control fibroblasts, in a similar profile to that previously found in human heart tissues (14).

### FRDA fibroblasts showed dysregulated acylcarnitine levels and PUFA enrichment

**Fig. 5:**
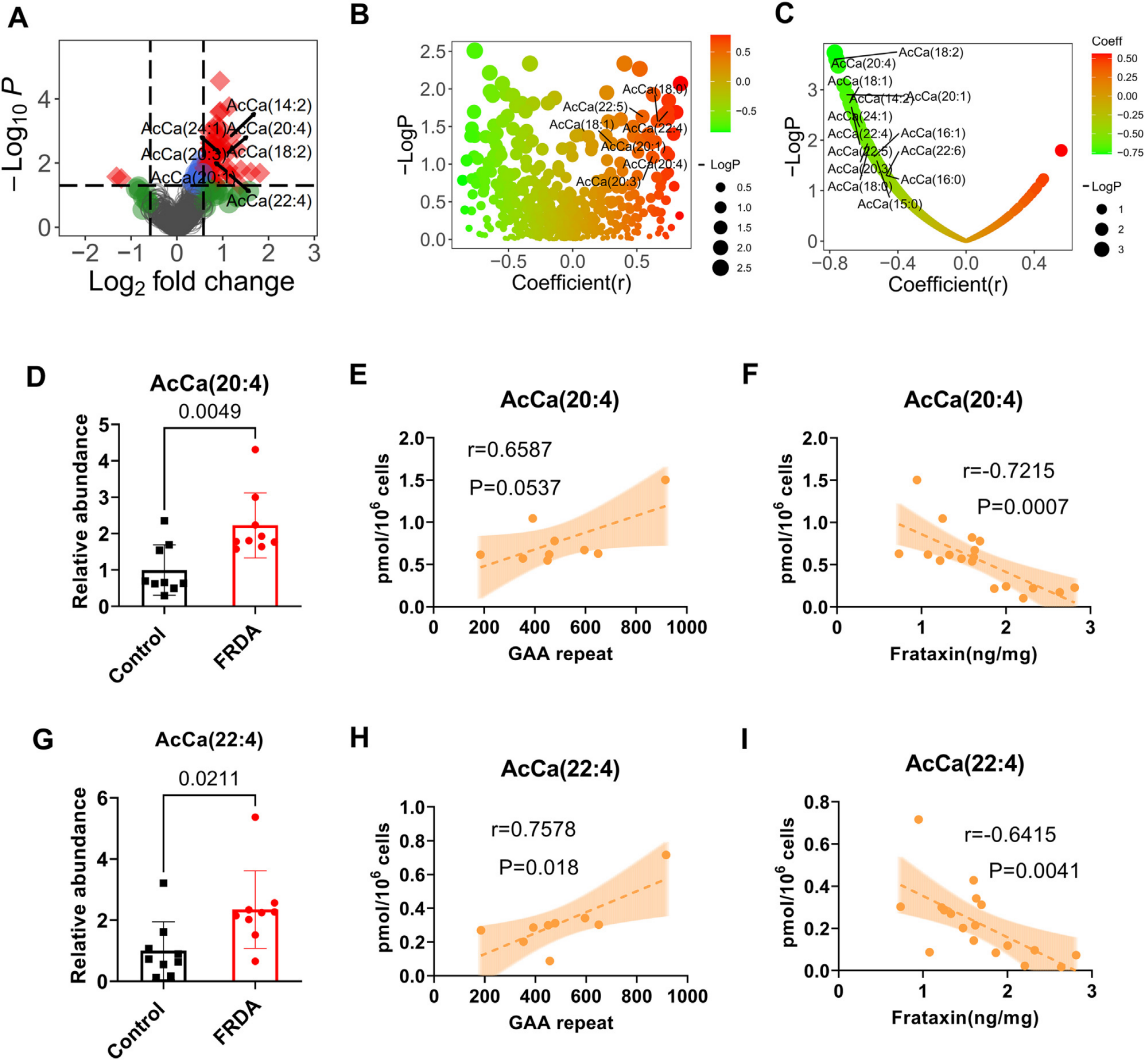
Acylcarnitines increased in FRDA fibroblast cells and correlated with GAA repeat length and frataxin levels. A: Volcano plot showing the dysregulated acylcarnitine, with most containing a PUFA. B: Pearson correlations between acylcarnitine levels and GAA repeat length. C: Pearson correlations between acylcarnitine levels and frataxin levels. (D The relative abundance of AcCa (20:4). E: Pearson correlation between AcCa (22:4) levels and GAA repeat. F: Pearson correlation between AcCa (22:4) levels and frataxin levels. G: The relative abundance of AcCa (22:4). H: Pearson correlation between AcCa (22:4) intensity and GAA repeat. I: Pearson correlation between AcCa (22:4) intensity and frataxin levels. All metabolites were quantified from 1∗E6 cells.

Several acylcarnitines, including AcCa (14:2), AcCa (18:2), AcCa (20:1), AcCa (20:3), AcCa (20:4), AcCa (22:4), and AcCa (24:1), were increased in FRDA fibroblasts (Fig. 5A), implying dysregulated fatty acid oxidation, complementing the increased HMG-CoA and BHB-CoA from the metabolomics profile and the platelets labeling data (11). Acylcarnitine levels positively correlated with GAA repeat length (Fig. 5B). Acylcarnitine levels negatively correlated with frataxin levels (Fig. 5C). For example, AcCa (20:4) was significantly increased in FRDA fibroblasts (Fig. 5D) and had a good correlation with GAA repeat length (r=0.6, *P*=0.076) (Fig. 5E), and a strong negative correlation was observed with frataxin levels (r=0.8, *P*=0.001) (Fig. 5F). Similar results were observed for AcCa (22:4) (Fig. 5G–I). Interestingly, most of the dysregulated acylcarnitines contained a polyunsaturated fatty acid (PUFA) chain.

### FRDA fibroblasts showed dysregulated TGs and other phospholipid levels with marked PUFA enrichment

**Fig. 6:**
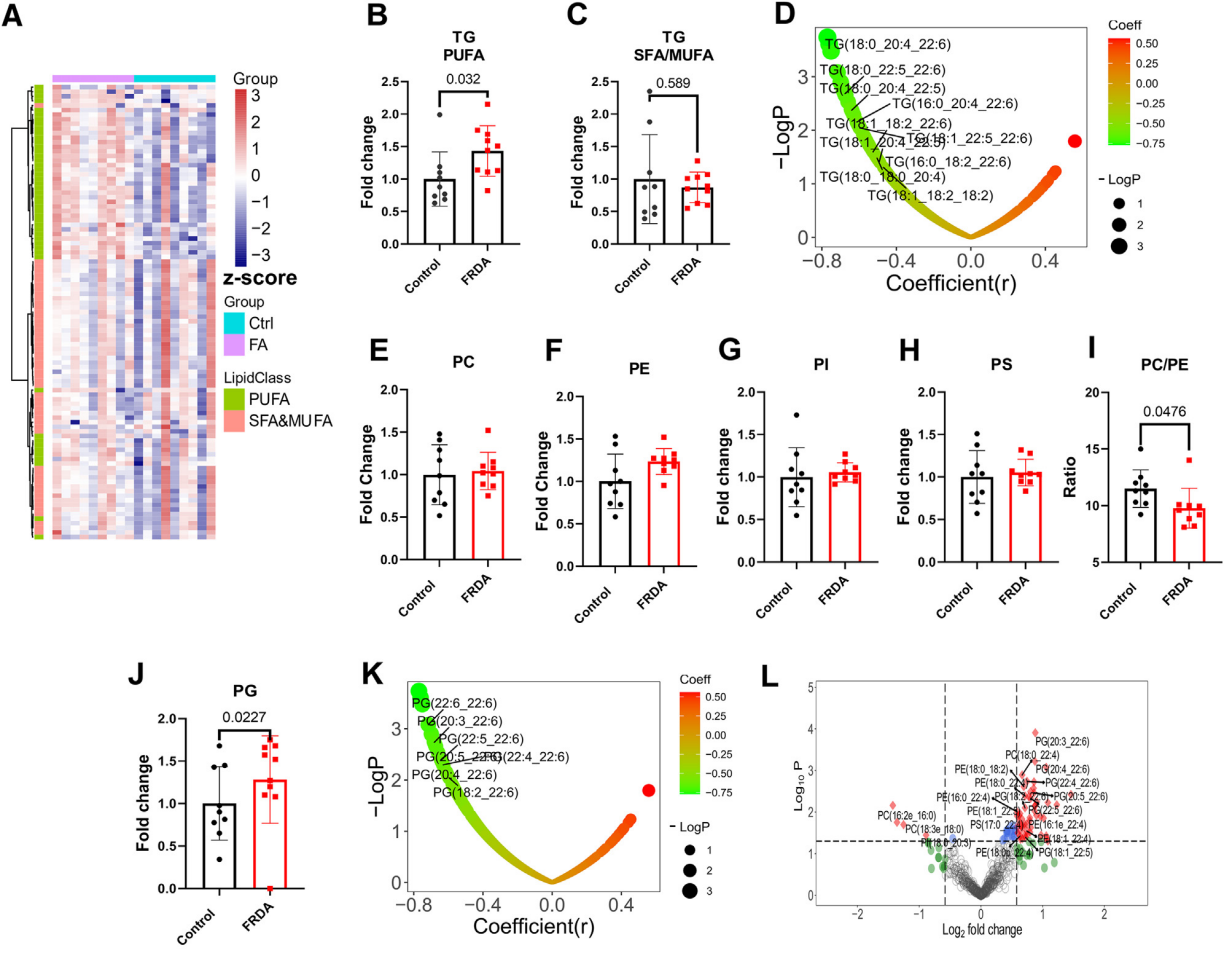
FRDA fibroblasts had dysregulated triglyceride and phospholipid metabolism. A: Heatmap showing only the TG in fibroblast cells, clustering based on unsaturation. The heatmap is presented as the z-scores (mean centered and divided by standard deviation) of each feature. Changes in (B) PUFA-TG and in (C) SFA/MUFA-TG in Control and FRDA fibroblast cells. D: Pearson correlation between TG and frataxin levels. E–H, Fold changes in the levels of PC, PE, PI, and PS. I: PC/PE ratio. J: PG levels in control and FRDA fibroblast cells. (K) Pearson correlation between PG and frataxin levels. (L) Volcano plots showing the significantly dysregulated phospholipids. Abbreviation: SFA, saturated fatty acid;. All metabolites were quantified from 1∗E6 cells.

The enrichment of PUFA in lipids was also observed in TGs composition (Fig. 6A). PUFA-TGs levels were significantly increased in FRDA fibroblasts (Fig. 6B), while levels of saturated fatty acids (SFAs) and monounsaturated fatty acids (MUFAs)-TGs were unaffected by the frataxin deficiency (Fig. 6C). Moreover, several of the PUFA-TGs levels negatively correlated with frataxin levels (Fig. 6D). The levels of most PUFA-TGs were increased 1.5-3× in FRDA compared with the control fibroblasts. Interestingly, even if the overall levels of total PCs and PEs species (also phosphatidylinositols and PSs) were not significantly different between groups (Fig. 6E–H), the ratio of total PCs to total PEs species, a good index of cellular methylation status (32) was significantly decreased in FRDA fibroblast compared with controls (Fig. 6I).

Similarly with the levels of TGs, the PGs levels were significantly increased in FRDA fibroblasts (Fig. 6J); most of these PGs contained PUFA. Levels of the PGs negatively correlated with frataxin levels (Fig. 6K). All other significantly changed phospholipids also showed PUFA enrichment (Fig. 6L).

## Discussion

The data from the fibroblast cells showed that the dysregulation in lipid metabolism extends in patients with FRDA to unaffected tissues (fibroblasts). Our results regarding increased ceramides in FRDA fibroblast cells replicate the findings from the heart tissue (14) of patients with FRDA and extend them by showing clear correlation with disease severity and implying a correlation with underlying disease mechanism.

Ceramides are one of the most studied classes of bioactive sphingolipids, as they regulate stress resistance, proliferation, differentiation, and maturation of different cells (33). Local or global dysregulation of ceramide levels can affect the cellular biology at multiple levels, thus contributing to disease pathogenesis (33). Cer de novo synthesis happens in the endoplasmic reticulum from palmitoyl-CoA and serine, using four enzymes; this process relies heavily on the availability of other acyl-CoA for the N-acetylation of sphinganine (Fig. 4A) by ceramide synthase (CerS). To date there are six different CerS known (supplemental Table S3), each with a tissue and substrate specificity (34). A recent study (35) in cardiomyocytes showed that overexpression of CerS2 elevated levels of very-long-chain ceramides and caused insulin resistance, oxidative stress, mitochondrial dysfunction, and mitophagy, suggesting a similar mechanism in cardiomyocytes from patients with FRDA. Palmitate induced CerS2 and oxidative stress, mitophagy, and apoptosis, which were prevented by depletion of CerS2. Increased oxidative stress has been proposed to contribute to the pathogenesis of Friedreich’s ataxia and could lead to the activation of Cer pathway. S-1-P opposes the proapoptotic role of Cer by decreasing oxidative stress and modulating the expressions of Bcl-2 family pro/anti-apoptotic proteins and the PI3K-Akt pathway (36) but there was no significant difference in the levels of S-1-P between the two groups (Fig. 6S). Ceramides affect the autophagic flux via Beclin1/Bcl-2 or mTOR and can induce changes in membrane fluidity and membrane trafficking (37). Our results showed different dysregulation between classical d18:0-Cer and d18:1-Cer, and d18:2-Cer, supporting the existing knowledge of a distinct biochemical pathway for regulating ceramides with different backbone structures (38).

Ceramides are believed to be a main factor in reorganizing microscopic membrane rafts into signaling platforms in response to stress. (39) Ceramides can induce apoptosis, increasing the risk for type 2 diabetes and cardiovascular disease, which fits with an involvement in the FRDA disease etiology (1). Combining results from the lipid profiling and isotope tracing experiments, our data from fibroblast cells indicate that frataxin deficiency enhances ceramide biosynthesis, contributing to the ceramide accumulation in the FRDA fibroblasts, while hydrolysis of more complex sphingolipids (Hex1Cer and SM) does not contribute to the ceramide accumulation (Fig. 5G). Given that skin shares a very similar expression profile of CerS (Fig. 4) with heart tissue (40), skin fibroblasts seem to be a good surrogate to study ceramide synthesis for heart disease, especially for biomarker discovery.

Acylcarnitines are important lipid biomarkers reflecting acyl-CoA status (41) and fatty acid oxidation (42, 43). The elevation of acylcarnitines in FRDA fibroblasts indicates an abnormal catabolism of lipids in these cells. Such fatty acid oxidation dysfunction has been observed in different FRDA models (44, 45, 46), usually concurrent with dramatic lipid accumulation (44, 46). HMG-CoA and BHB-CoA, as the catabolic products of fatty acids, were also significantly increased in FRDA fibroblasts, replicating findings in FRDA platelets (11). Although lipid metabolism has not been fully characterized in FRDA, fatty acid oxidation seemingly can serve as an adaptive compensatory mechanism to realize the energy demands and redox homeostasis. In a transgenic mouse model of inducible FXN depletion, β-oxidation was relatively preserved when pyruvate oxidation was blocked, which could allow maintenance of cardiac contractility (although no direct connection was demonstrated) (47). In neonatal rat ventricular myocytes, activation of PPARα-dependent pathways was observed after frataxin depletion, as fatty acid oxidation-related MCAD and CPT1 were increased (46). The activation of fatty acid oxidation was also observed in neuronal cells after Complex I inhibition, which supported acetyl-coenzyme A levels (48). All these previous observations combined with our findings of clearly elevated levels of acylcarnitines in FRDA fibroblast cells (Fig. 5) indicate that the lipid dysregulation is an extensive event in FRDA, reflected in unaffected tissues as well.

Apart from having the role as biomarkers, acylcarnitines also possess bioactive and inflammatory properties and can regulate the function of membrane-based systems and impact cell biology. (49) In cardiac ischemia, cardiac acylcarnitine accumulation has been associated with increased cardiac cellular reactive oxygen species production, and endoplasmic reticulum stress, apoptosis, and increases in intracellular calcium (50, 51, 52). It is worth investigating further if the elevation of acylcarnitine and catabolism products contribute to the pathogenesis of FRDA.

As mentioned, the metabolites BHB-CoA and HMG-CoA accumulated in FRDA fibroblast cells (Fig. 1G), but acetyl-CoA levels were not significantly disrupted, demonstrating a high compensatory ability of these cells to maintain a constant level of acetyl-CoA. Because fibroblasts are not directly involved in disease pathology, but recapitulate so well the increased fatty acid oxidation, this compensatory mechanism could be further investigated in combination with histone deacetylation inhibitors that increase frataxin gene expression (53) where we could monitor how acetyl-CoA levels are affected. Recent advances in FRDA research have revealed the presence of several epigenetic modifications, including hypoacetylation and hypermethylation that are involved in this FXN gene silencing (54, 55). The decreased ratio of PC to PE resulting from the lack of PE methylation could lead to the accumulation of S-adenosylmethionine (methyl donor) involved in the histone hypermethylation (32). Increased methylation of histone residues (H3K9me2/3 and H3K27me3) had been observed in other FRDA fibroblasts cell lines (55). Therefore, the ratio of PC to PE in fibroblasts may reflect the hypermethylation status in patients with FRDA. Further work will investigate the ratio of S-adenosylmethionine to S-adenosylhomocysteine, a known inhibitor of methyltransferases.

HMG-CoA serves as the precursor to isoprenoid groups that are incorporated into a wide variety of end-products, including cholesterol, ubiquinone, heme, and dolichol. Our lipidomic profiling showed that two important products of HMG-CoA, CoQ10 and cholesterol, are not affected by frataxin deficiency, which may explain why improvements by idebenone (an analogue of CoQ10) were not statistically significant in clinical trials (56). Several studies have reported alteration in the heme pathway and decreased heme A levels in mammalian cells in FRDA (57, 58). As the precursor of heme A, the accumulation of HMG-CoA may result from defective heme A synthesis. HMG-CoA is also responsible for the generation of ketone bodies, metabolites that have not been investigated in this study. Altered expression of the HMGCL gene as determined from transcriptomic data indicated that ketogenesis may be influenced by frataxin deficiency (26). Considering the functions of ketone bodies as a mobile source of energy and inhibitor of histone deacetylases (59), further studies should be done to clarify if ketone bodies have roles in modulating frataxin gene expression and energy metabolism in FRDA models.

Notably, PUFAs were enriched in phospholipids and triglycerides (both elevated in FRDA fibroblasts) (Fig. 6), which would render the FRDA fibroblasts more sensitive to lipid peroxidation, consistent with increased oxidative stress (60) and iron levels in FRDA (12, 27). The redistribution of PUFA into triglycerides may serve as a reservoir to protect the cell membrane from ferroptosis, which has been observed in FRDA models (61). The PUFA dysregulation could be more severe in insolated mitochondria.

## Conclusion

Altogether, our work demonstrates that the levels of ceramides and acylcarnitines, directly correlate with disease severity in FRDA, making them possible biomarkers of disease, as accurate assays to quantify small molecules and lipids are less labor intensive than assays for accurate protein quantification. Furthermore, the isotope tracing experiment suggested the mechanism for ceramide accumulation, from de novo biosynthesis rather than complex lipid turnover. Currently there are several US Food and Drug Administration-approved drugs targeting ceramides synthesis that are extensively detailed by Kovilakath and Coward (15). Together with previous findings on lipids in FRDA, our work suggests that lipid accumulation affects all tissues and could be a secondary toxicity mechanism affecting membrane integrity early on. Our new finding related to dysregulated acylcarnitines in FRDA has the potential of a novel class of biomarkers for FRDA and could provide insights into the mechanism of pathophysiology (defective mitochondrial lipid oxidation, especially in heart) (62, 63). Finally, our results indicate that fibroblasts are a good surrogate tissue for biomarker discovery in FRDA. The possibility to collect FRDA fibroblast cells with GAA repeats that cover a large span of disease severity make them a promising tool for future biomarker studies in FRDA.

## Data availability

All data are contained within the article or supplemental material. Raw mass spec file data will be shared upon request from the corresponding author, Clementina Mesaros, PhD, Center of Excellence in Environmental Toxicology, Department of Systems Pharmacology and Translational Therapeutics, University of Pennsylvania, e-mail: mesaros@upenn.edu.

## Supplemental data

The Supporting Information is available free of charge.This article contains supplemental data (64).

## Conflict of interest

The authors declare that they have no conflict of interest with the contents of this article.
