# Supplementary material for: Skin fibroblast metabolomic profiling reveals that lipid dysfunction predicts the severity of Friedreich’s ataxia
Source: J Lipid Res. 2022 Jul 16;63(9):100255. doi: 10.1016/j.jlr.2022.100255 (PMC9399481; doi:10.1016/j.jlr.2022.100255)
Supplement: Supplementary Information [file mmc1.docx]

**Table S1.** Molecular and clinical data for FRDA and control fibroblast samples used for metabolomic and lipidomic analysis

| **Cell Line** | **Gender** | **Sampling Age (years)** | **GAA Repeat Lengths** | | **Age of Disease Onset (years)** |
| --- | --- | --- | --- | --- | --- |
|  |  |  | **Allele 1** | **Allele 2** |  |
| **FRDA** | | | | | |
| 203 | F | 31 | 916 | 1382 | 14 |
| *4111** | F | 19 | 392 | 485 | 12 |
| 4193 | F | 20 | 650 | 719 | 12 |
| 6247 | F | 33 | 478 | 1017 | 15 |
| 4687 | F | 23 | 450 | 1270 | 10 |
| 4182 | M | 22 | 457 | 684 | 11 |
| 4675 | M | 28 | 185 | 1130 | 4 |
| *4249** | M | 19 | 596 | 596 | 10 |
| 50 | M | 33 | 353 | 616 | 13 |
| **Mean** |  | **25.3** | **497.4** | **877.7** | **11.2** |
| **S.D.** |  | **5.6** | **194.9** | **308.4** | **3.0** |
| *4078** | M | 30 | 341 | 480 | ND |
| **Control** | | | | | |
| 7522 | F | 19 | N/A | N/A | N/A |
| 3956 | F | 27 | N/A | N/A | N/A |
| 2036 | F | 11 | N/A | N/A | N/A |
| 2671 | F | 47 | N/A | N/A | N/A |
| 7492 | M | 17 | N/A | N/A | N/A |
| 3348 | M | 10 | N/A | N/A | N/A |
| 2153 | F | 40 | N/A | N/A | N/A |
| 3652 | M | 24 | N/A | N/A | N/A |
| 2169 | M | 52 | N/A | N/A | N/A |
| **Mean** |  | **27.4** |  | | |
| **S.D.** |  | **15.5** |  |  |  |

**Lines used for the isotopic labeling experiment*

**Table S2.** Internal Standards (ISTD) added to each sample during metabolomic and lipidomic fibroblast extraction

| **Internal Standard (ISTD)** | **Concentration in Mix (ng µL^-1^)** | **Added Amount (ng per plate)** | **Supplier** |
| --- | --- | --- | --- |
| **Metabolomics ISTD Mix** | | | |
| Glycine – [13]C2, [15]N | 11 | 440 | CIL |
| Serine – [13]C3, [15]N | 60 | 2400 | CIL |
| Isoleucine – [13]C6, [15]N | 10.5 | 420 | CIL |
| Thymidine – [15]N2 | 11.5 | 460 | CIL |
| Threonine – [13]C4, [15]N | 11 | 440 | SA |
| Pyruvate – [13]C3 | 10 | 400 | CIL |
| Arginine – [13]C6 | 9.5 | 380 | CIL |
| Valine – [13]C5, [15]N | 110 | 4400 | CIL |
| Tryptophan – [13]C11 | 11.5 | 460 | CIL |
| Lysine – [13]C6 | 10 | 400 | CIL |
| Aspartic Acid – [13]C4, [15]N | 10.5 | 420 | CIL |
| Glutamic Acid – [13]C5, [15]N | 10 | 400 | SA |
| Glutamine – [13]C5, [15]N | 10 | 400 | SA |
| Gluconolactone – [13]C6 | 50 | 2000 | TRC |
| D-3-Hydroxybutyrate – [13]C4 | 10.5 | 420 | CIL |
| Adenosine – [13]C5 | 50 | 2000 | CIL |
| Ketoglutarate – D5 | 2.5 | 100 | CIL |
| Hydroxyglutarate – [13]C5 | 2.5 | 100 | SA |
| Oxalic Acid – [13]C2 | 5 | 200 | TRC |
| Fumaric Acid – [13]C4 | N/A | N/A | CIL |
| Succinic Acid – [13]C4 | 11 | 440 | SA |
| Malonic Acid – [13]C3 | 0.5 | 20 | TRC |
| Fructose 6-Phosphate – [13]C6 | 5 | 200 | TRC |
| Citric Acid – [13]C6 | 9 | 360 | SA |
| Fructose 1,6-Bisphosphate – 13]C6 | 12.5 | 500 | TRC |
| Acetyl-CoA – [13]C2 | 2.5 | 100 | SA |
| Lactate – [13]C3 | 80 | 3200 | CIL |
| GSH – [13]C2, [15]N | 50 | 2000 | CIL |
| Adenosine-monophosphate – [13]C10, [15]N5 | 362.08 | 14483 | SA |
| Deoxyadenosine-monophosphate – [13]C10, [15]N5 | 344.07 | 13763 | SA |
| Adenosine-triphosphate – [13]C10, [15]N5 | 522.01 | 20881 | SA |
| Deoxyadenosine-triphosphate – [13]C10, [15]N5 | 506.02 | 20241 | SA |
| Thymidine-triphosphate – [13]C10, [15]N2 | 494.02 | 19761 | SA |
| Guanosine-monophosphate – [13]C10, [15]N5 | 378.08 | 15123 | SA |
| Guanosine-triphosphate – [13]C10, [15]N5 | 538.01 | 21520 | SA |
| Cytidine-triphosphate – [13]C9, [15]N3 | 495.01 | 19800 | SA |
| Uridine-triphosphate – [13]C9, [15]N2 | 494.99 | 19800 | SA |
| ^1^Carnitine – D9 | 13.8 | 552 | CIL |
| ^1^Acetylcarnitine – D3 | 3.79 | 151.71 | CIL |
| ^1^Propionylcarnitine – D3 | 0.81 | 32.58 | CIL |
| ^1^Butyrylcarnitine – D3 | 0.85 | 34.19 | CIL |
| ^1^Isovalerylcarnitine – D9 | 0.94 | 37.62 | CIL |
| ^1^Octanoylcarnitine – D3 | 1.04 | 41.79 | CIL |
| ^1^Myristoylcarnitine – D9 | 1.39 | 55.53 | CIL |
| ^1^Palmitoylcarnitine – D3 | 3.02 | 120.71 | CIL |
| **Lipidomics ISTD Mix** | | | |
| ^2^15:0-18:1 PC – D7 | 80 | 1600.0 | APL |
| ^2^15:0-18:1 PE – D7 | 2.5 | 50.0 | APL |
| ^2^15:0-18:1 PS – D7 | 2.5 | 50.0 | APL |
| ^2^15:0-18:1 PG – D7 | 15 | 300.0 | APL |
| ^2^15:0-18:1 PI – D7 | 5 | 100.0 | APL |
| ^2^15:0-18:1 PA – D7 | 3.5 | 70.0 | APL |
| ^2^18:1 LPC – D7 | 12.5 | 250.0 | APL |
| ^2^18:1 LPE – D7 | 2.5 | 50.0 | APL |
| ^2^18:1 Cholesterol Ester – D7 | 175 | 3500.0 | APL |
| ^2^18:1 MG – D7 | 1 | 20.0 | APL |
| ^2^15:0-18:1 DG – D7 | 5 | 100.0 | APL |
| ^2^15:0-18:1-15:0 TG – D7 | 27.5 | 550.0 | APL |
| ^2^18:1 SM – D9 | 15 | 300.0 | APL |
| ^2^Cholesterol – D7 | 50 | 1000.0 | APL |
| 5-HETE – D8 | 5 | 100.0 | CC |
| Sphingosine (C17 base) | 3.6 | 71.3 | APL |
| Sphinganine (C17 base) | 3.6 | 71.8 | APL |
| Sphingosine-1-P (C17 base) | 4.6 | 91.3 | APL |
| Sphinganine-1-P (C17 base) | 4.6 | 91.8 | APL |
| Lactosyl(ß) C12 Ceramide | 8.1 | 161.6 | APL |
| 12:0 Sphingomyelin | 6.0 | 120.4 | APL |
| Glucosyl(ß) C12 Ceramide | 8.0 | 160.9 | APL |
| 12:0 Ceramide | 10.1 | 201.4 | APL |
| 12:0 Ceramide-1-P | 7.0 | 140.4 | APL |
| 25:0 Ceramide | 8.3 | 165.9 | APL |

CIL = Cambridge Isotope Laboratories, Inc., SA = Sigma-Aldrich, TRC = Toronto Research Chemicals, Inc., APL = Avanti Polar Lipids, Inc., CC = Cayman Chemicals

^1^ Labeled carnitine standards set B from CIL

^2^ Splash Lipidomix Mass Spec Standards from APL

**Table S3.** Ceramide Synthase: tissue location and substrate specificity.

| **CerS** | **Acyl-Chain Length Specificity** | **Tissue mRNA Expression Profile** |
| --- | --- | --- |
| CerS1 | C18 | Brain, skeletal mm, testis |
| CerS2 | C20-C26 | Kidney, Liver |
| CerS3 | C22-C26 | Testis, Skin |
| **CerS4** | **C18-C20** | Low expression all tissues (more in skin, leukocytes, heart, liver) |
| CerS5 | C16 | Low expression in all tissues |
| CerS6 | C14, C16 | Low expression in all tissues |


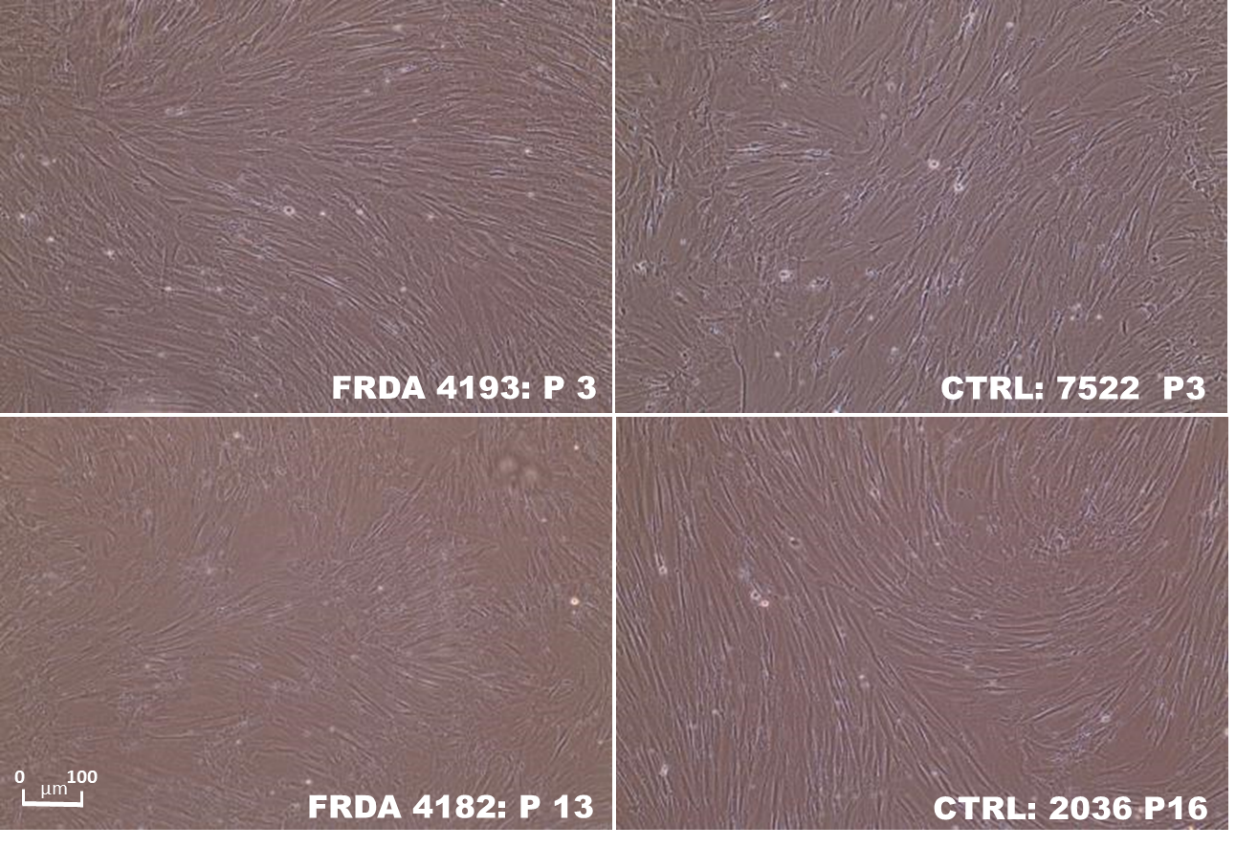


**Figure S1:** FRDA fibroblast looked similar with healthy control fibroblast under the microscope (10x zoom). The lowest passage number for FRDA line and healthy controls was passage 3 (top panels). The highest passage number was P16 for controls and passage 13 for FRDA fibroblast (lower panels). All cells were adjusted to 1 million cells per plate and did not have significant difference in the time to reach the 1 million cells.


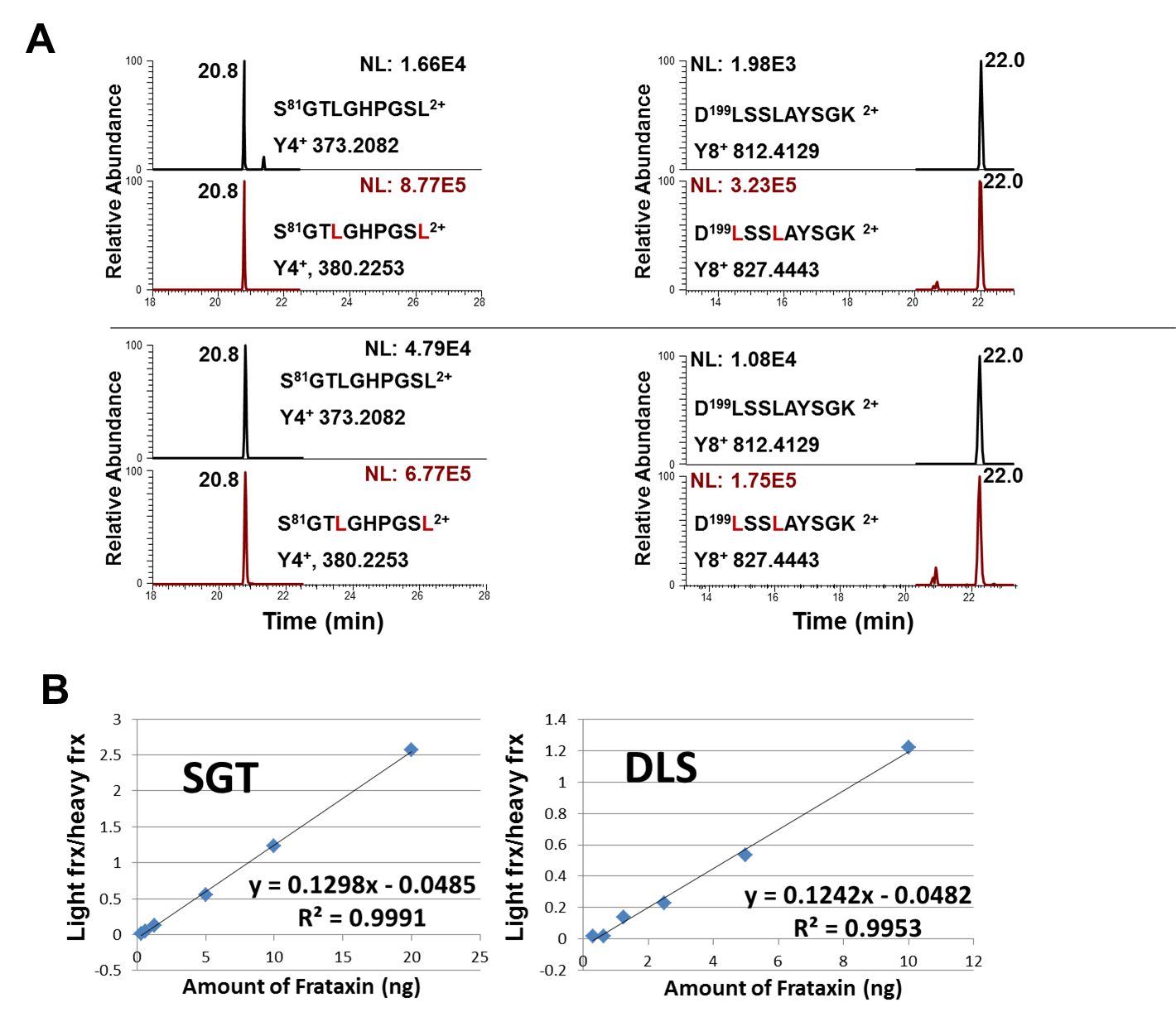


**Figure S2.** Typical chromatograms of AspN digestion peptide for quantification of frataxin protein from fibroblast cells. S81GTLGHPGSL2+ is the peptide used for mature form of frataxin quantification and D199LSSLAYSGK 2+ is used for the total frataxin quantification. Heavy leucine labeled frataxin was used as an internal standard. generated peptide. (A) Top panel are peptides from a FRDA fibroblast cell line and bottom panel is from a control fibroblast cell line.(B) Calibration curves constructed with authentic human frataxin and same amount of labeled frataxin as was added for the fibroblast cells.

**Figure S3.** Quantification of glycolysis metabolites in Control and FRDA fibroblasts.


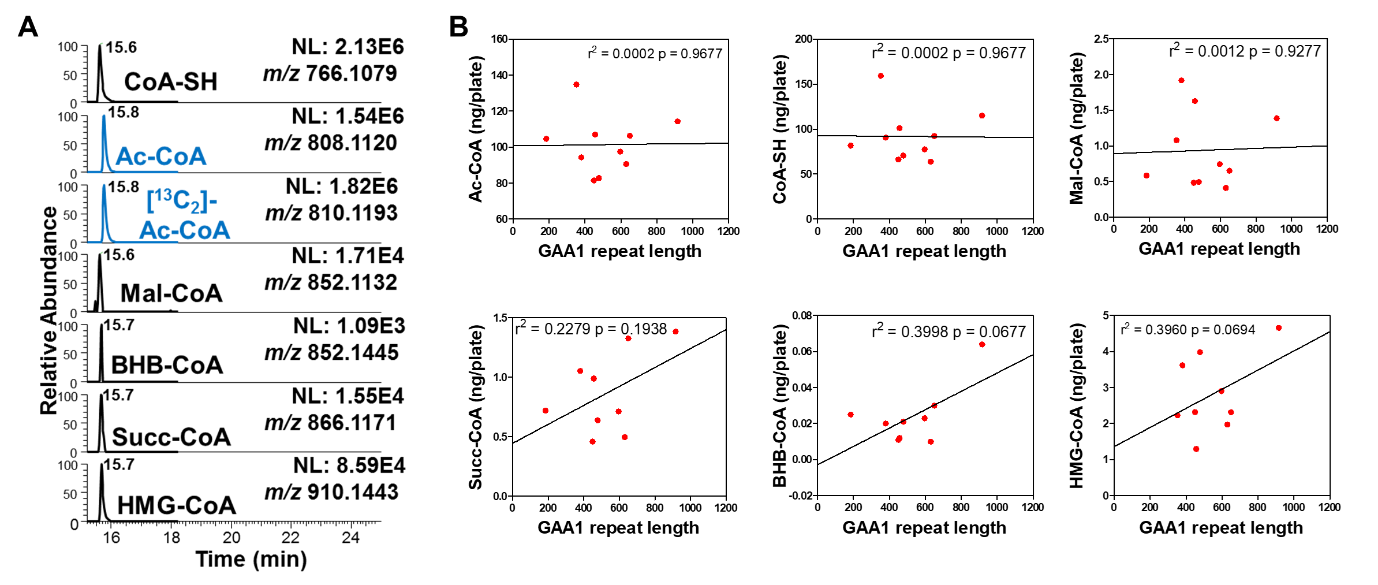


**Figure S4.** (A) Chromatograms constructed with authentic standards and stable isotope internal standards. (B) No significant correlation was observed between the levels of short-chain-CoAs and the GAA repeat of the FRDA fibroblasts. HMG-CoA and BHB-CoA were the only ones that showed a weak correlation (r2=0.39 and r2=0.39) and close to reaching significant value (p=0.069 and p=0.067).


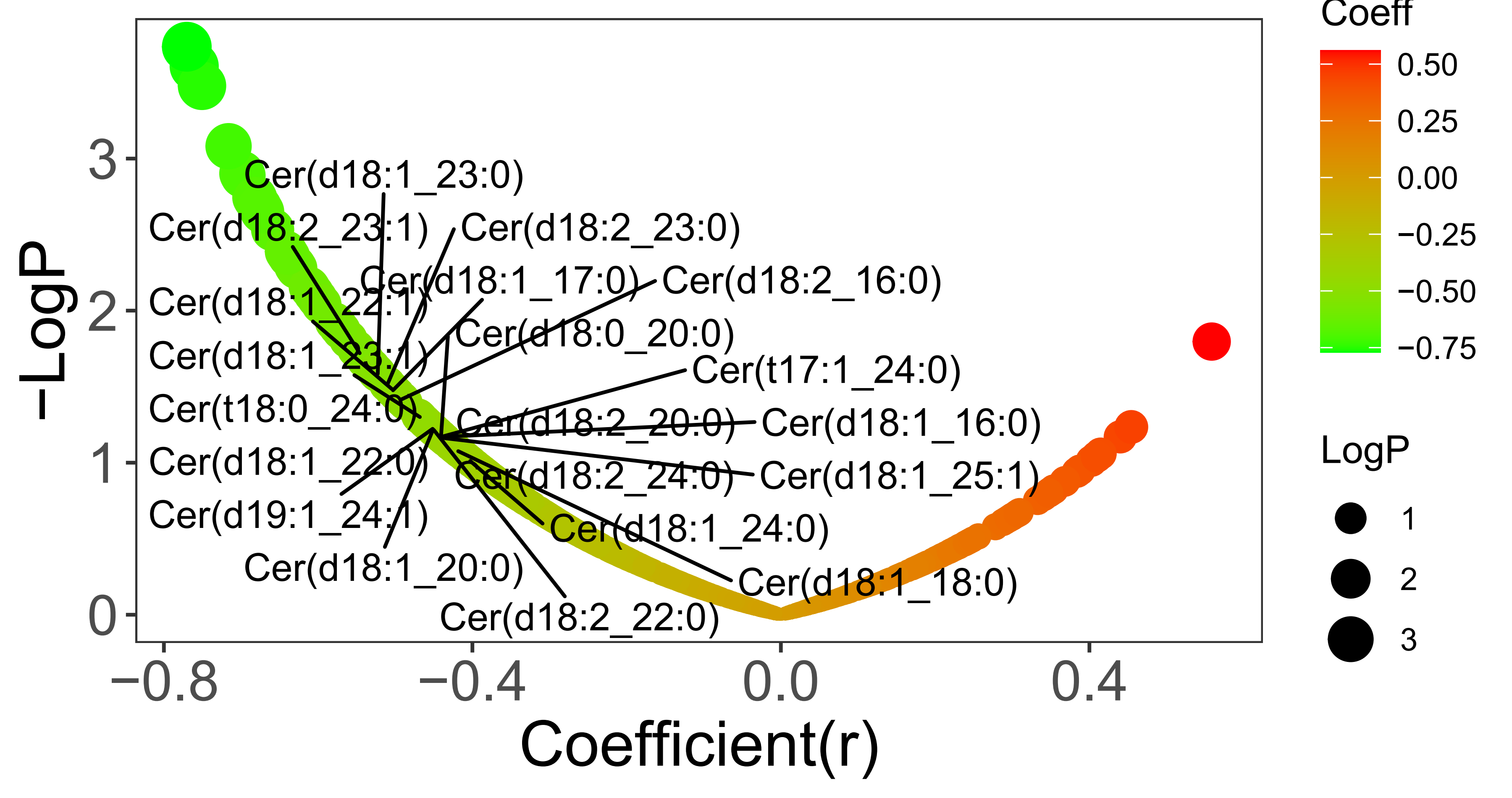


**Figure S5.** Pearson correlation between ceramides and frataxin levels. Ceramides correlated with frataxin levels with p value less than 0.1.


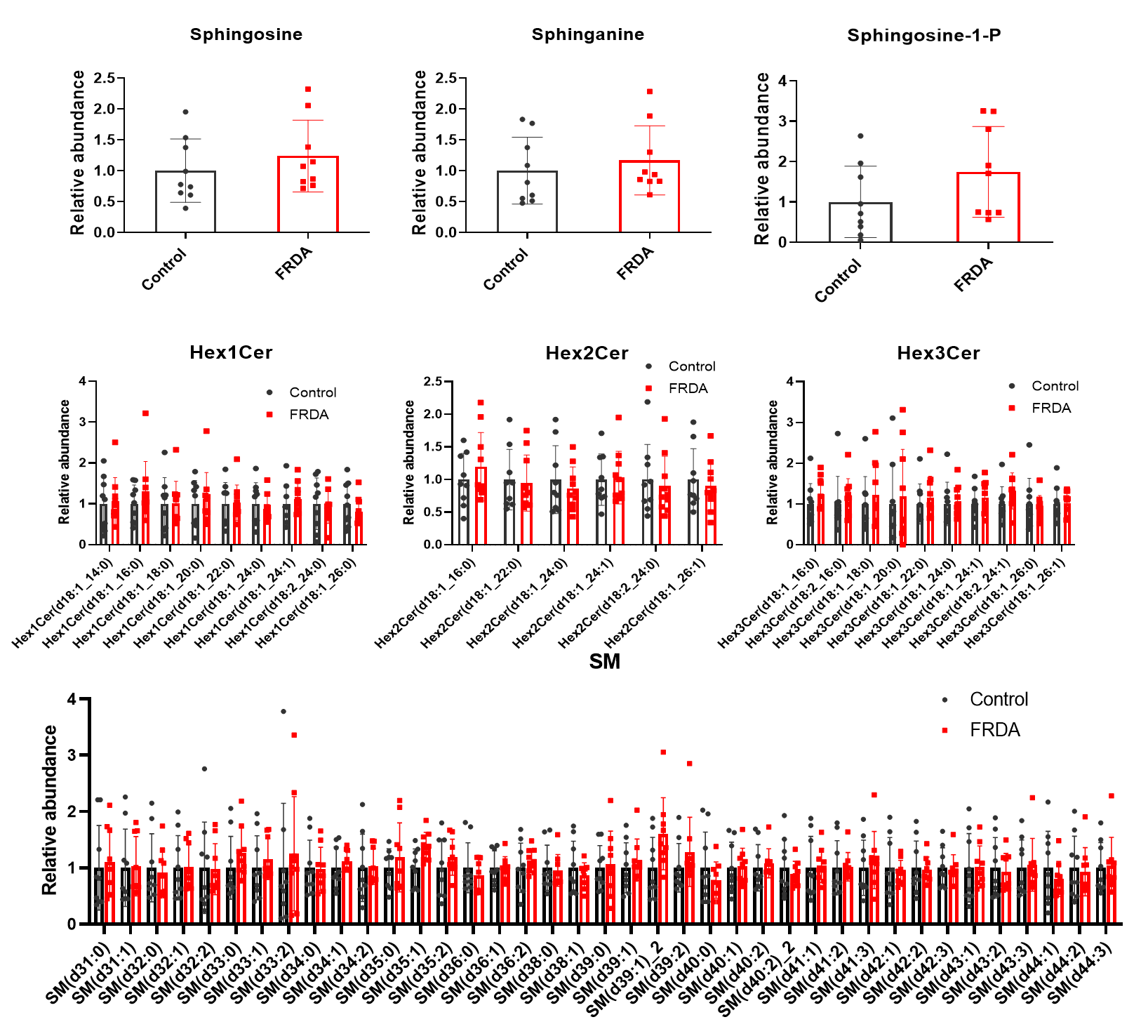


**Figure S6.** The relative abundance of sphingolipids, including sphingosine, sphinganine, sphingosine-1-P, Hex1Cer, Hex2Cer, Hex3Cer and SM in Control and FRDA fibroblasts.


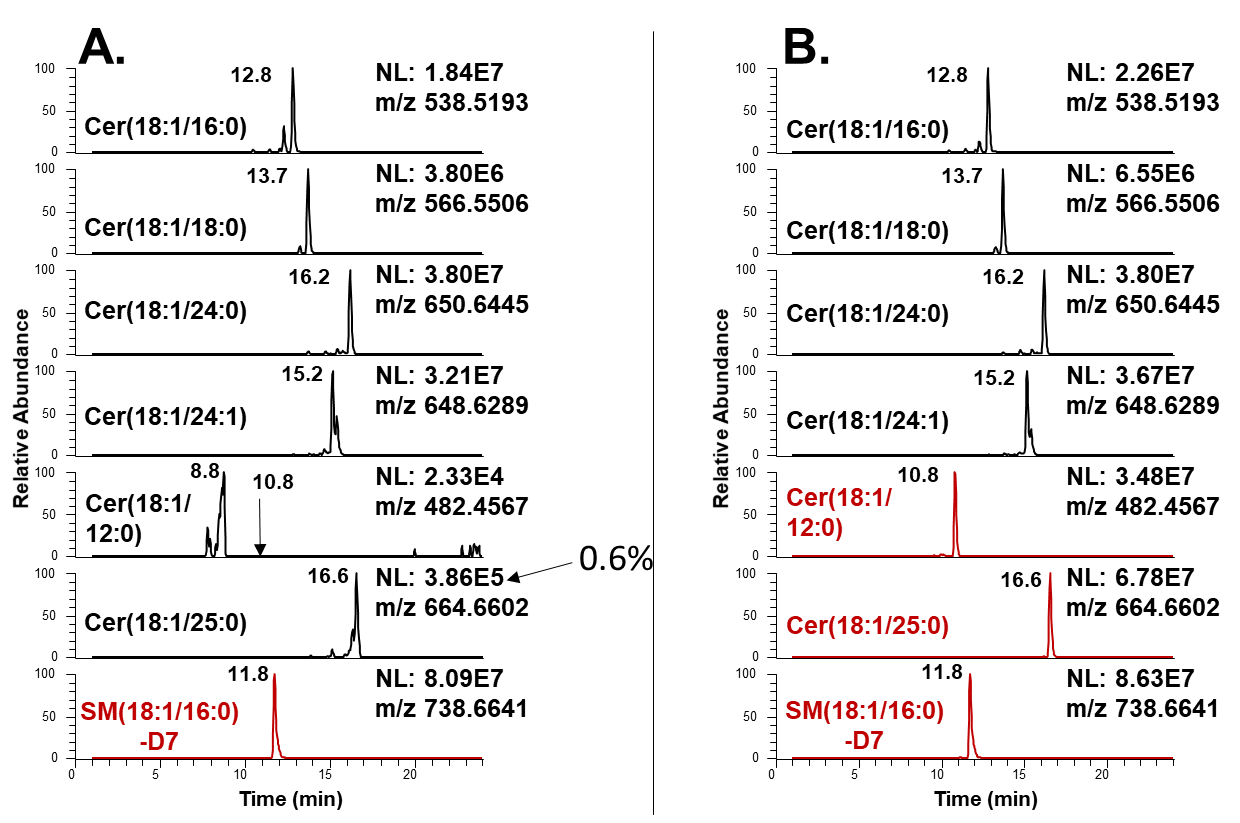


**C.**

**
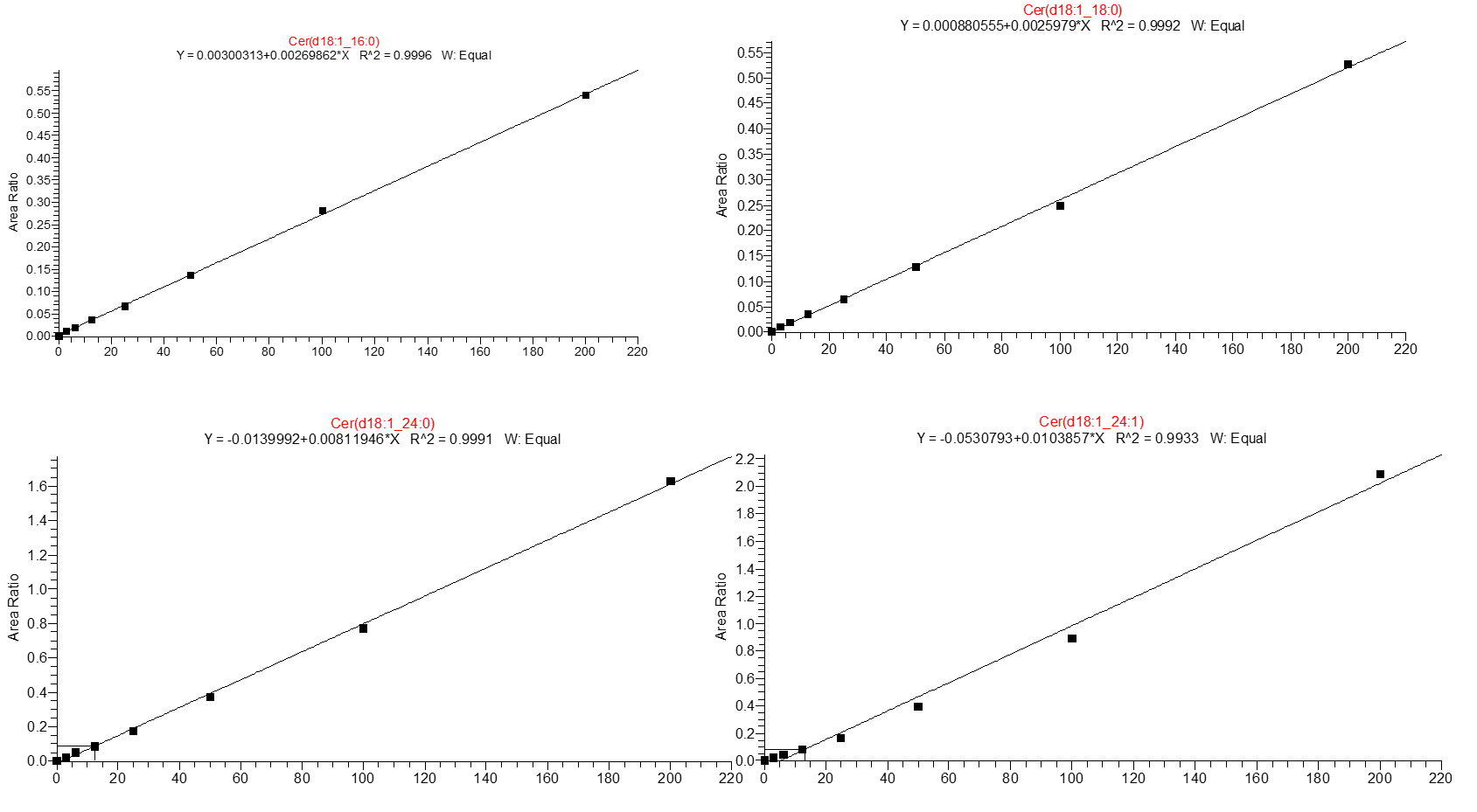
**

**Figure S7.** Chromatograms from 1 million control fibroblast cells extracted by same method but with different internal standard mixutres. A. extracted after spiked with Splash Lipidomix (10 µL stock from Avanti), when only SM-D7 can be used as internal standard (IS). Cer (18:1/12:0) has no visible peak at ret time 10.8 min and Cer (18:1/25:0) has a peak area that is about 0.6% from the same peak in panel B. B. extracted after spiked with Splash Lipidomix (10 µL stock from Avanti) and Ceramide/Sphingoid Internal Standard Mixture I ((10 µL stock from Avanti). Both Cer (18:1/12:0) and Cer (18:1/25:0) showed peaks with intensities in the same range as the endogenous ceramides from fibroblast cells. C. Calibration curves from two long chain ceramides and two very long ceramides were linear in the range comparable with the levels of ceramides from the cells (area ratio around 1).


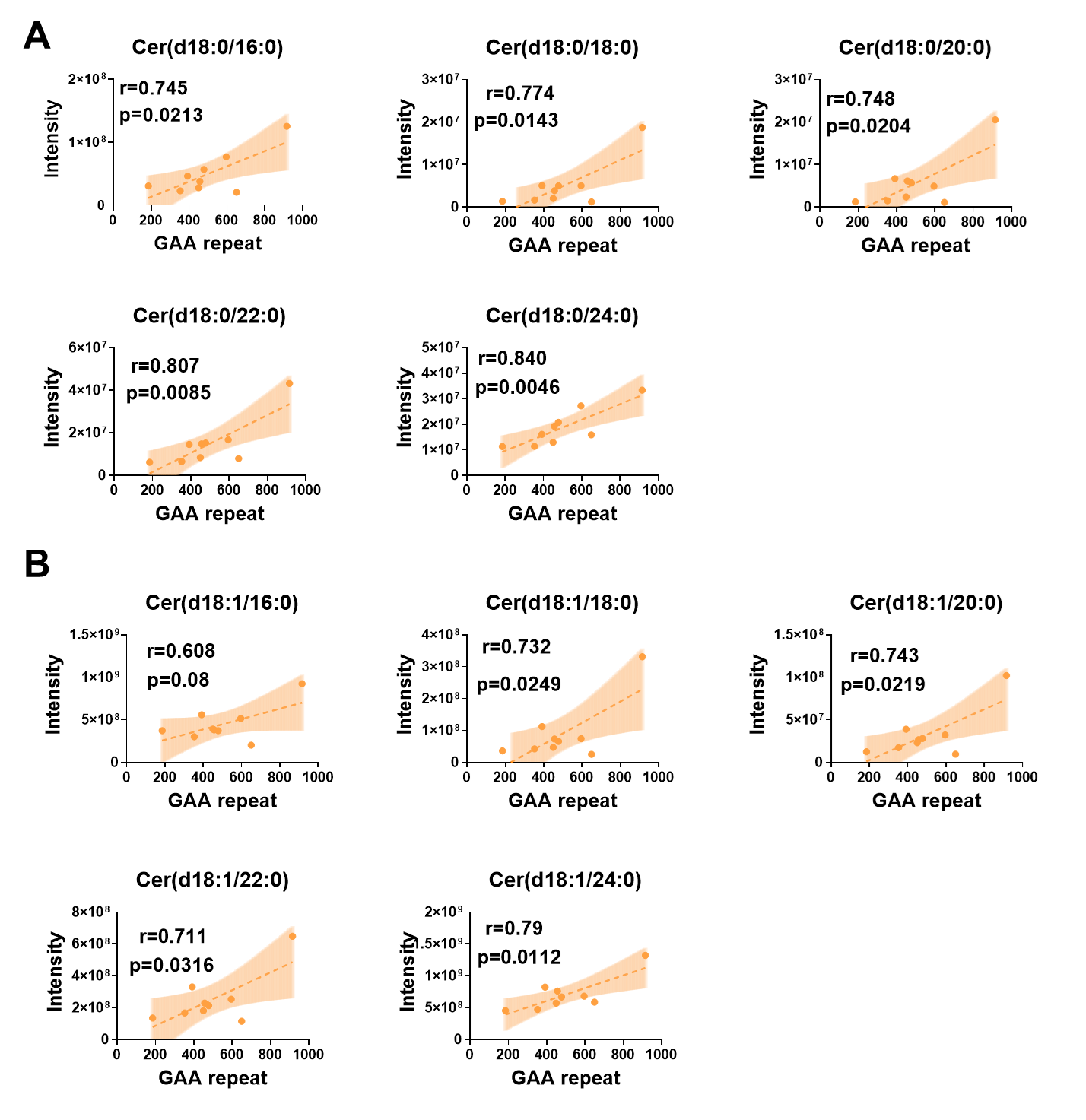


**Figure S8.** Pearson correlation plots between (A) dihydroceramides (d18:0) or (B) ceramides (d18:1) and GAA repeat length.

**Figure S9.** The correlation plots between ceramide(d18:1) and frataxin levels.


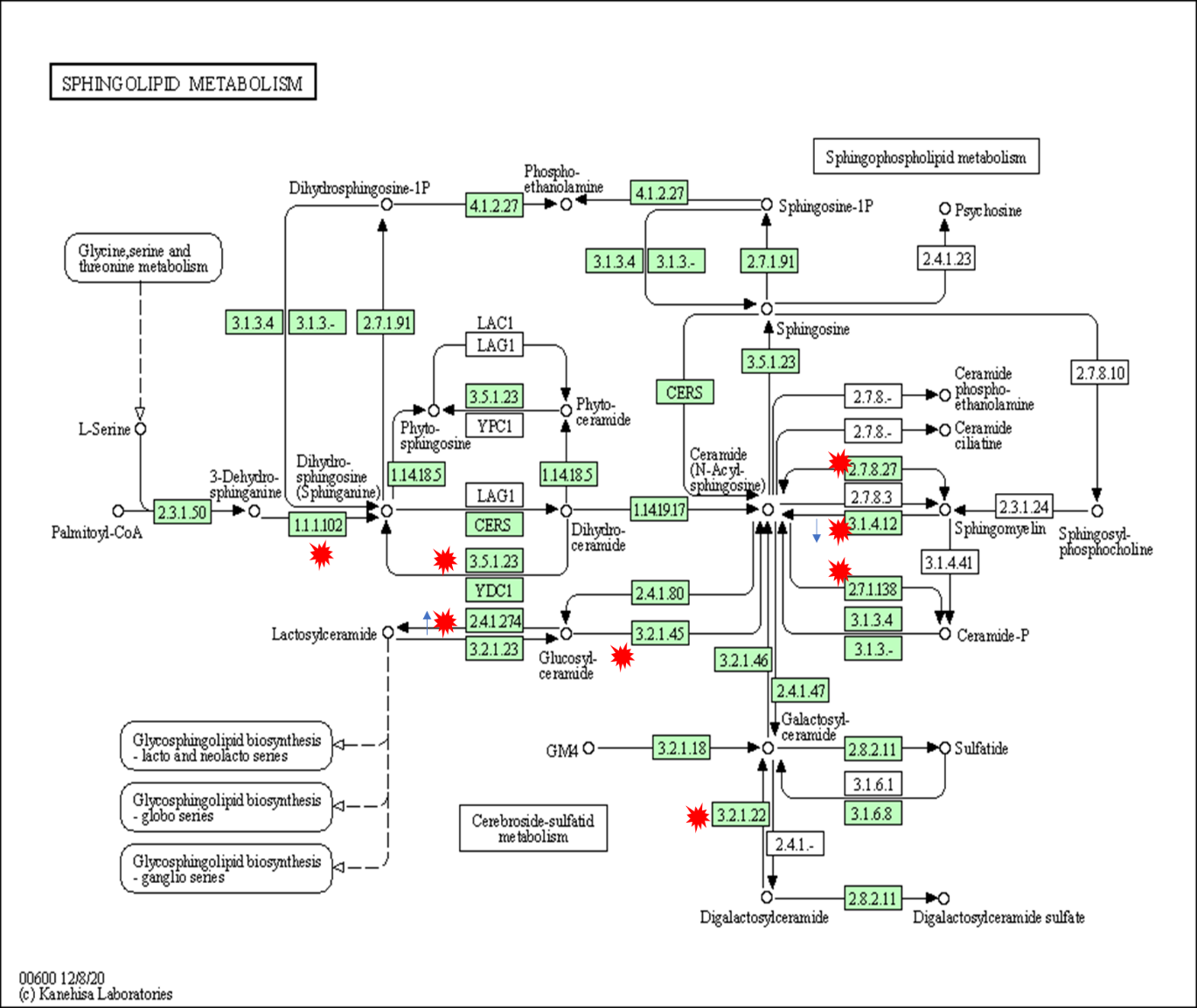


**Figure S10**. The analysis of the transcriptomic data identified several transcripts that were highly dysregulated (p-values < 0.01; FDR <0.050 and could be mapped to the “Sphingolipid metabolism” (KEGG pathway map hsa 00600).


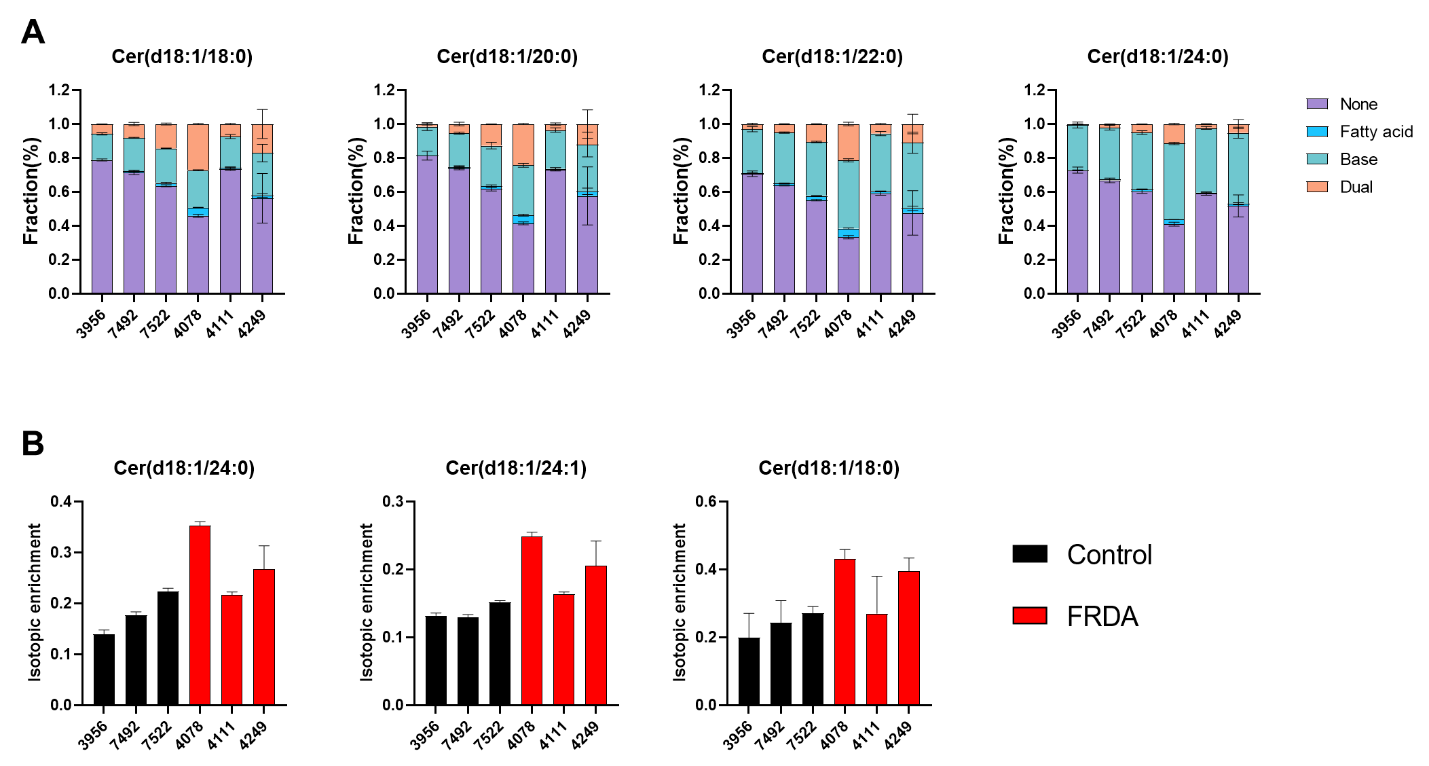


**Figure S11.** (A) Mass isotopomer distribution of ceramides. (B) The isotopic enrichments of Ceramides**.**


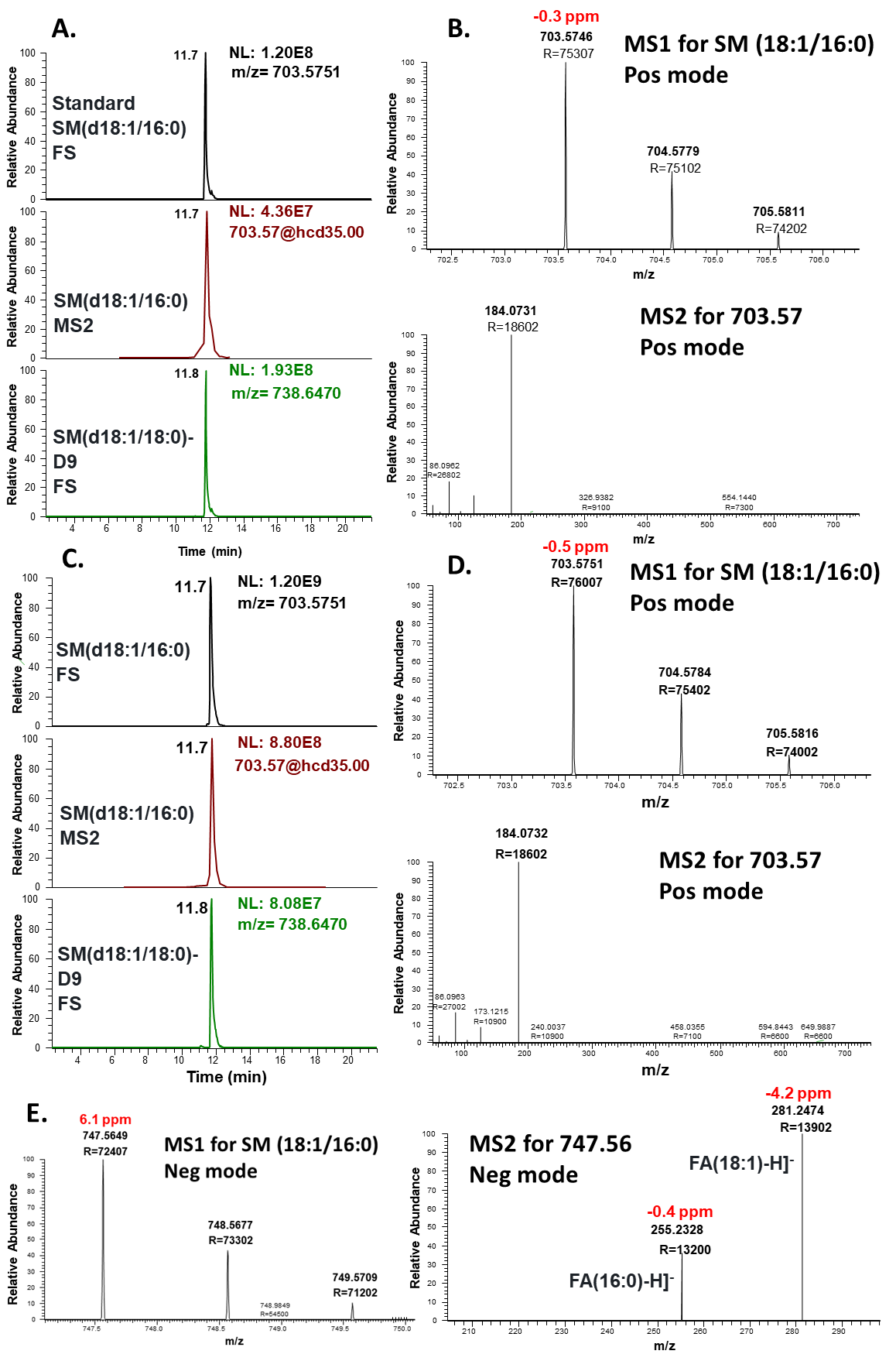


**Figure S12.** (A) Chromatogram of 200 ng of SM(d18:1/16:0) and d9-SM(d18:1/18:0 (both from Avanti Lipids). (B) MS1 of the standard SM(d18:1/16:0) matched the predicted mass with 0.3 ppm. (top panel). MS2 in positive mode (lower panel) showed the characteristic choline head loss from SM. (C) Chromatogram from 1 million of fibroblast cells control showing the SM(d18:1/16:0) and internal standard d9-SM(d18:1/18:0. ). (D) MS1 of the peak corresponding to the SM(d18:1/16:0) matched the predicted mass with 0.5 ppm. (top panel). MS2 in positive mode (lower panel) showed the characteristic choline head loss from SM, exactly as in the case of the authentic standard SM(d18:1/16:0). (E) using the negative mode for the fibroblast sample the chromatographing peak at 11.8 min showed the MS1 corresponding to the negative ion [M+HCOO]^-^ of SM(d18:1/16:0) with 6.1 ppm from the theoretical mass (left panel) and the MS2 of 747.56 (right panel) showed the two fatty acid chain corresponding to FA(16:0)-1]^-^ and FA(18:1)-1]^-^.


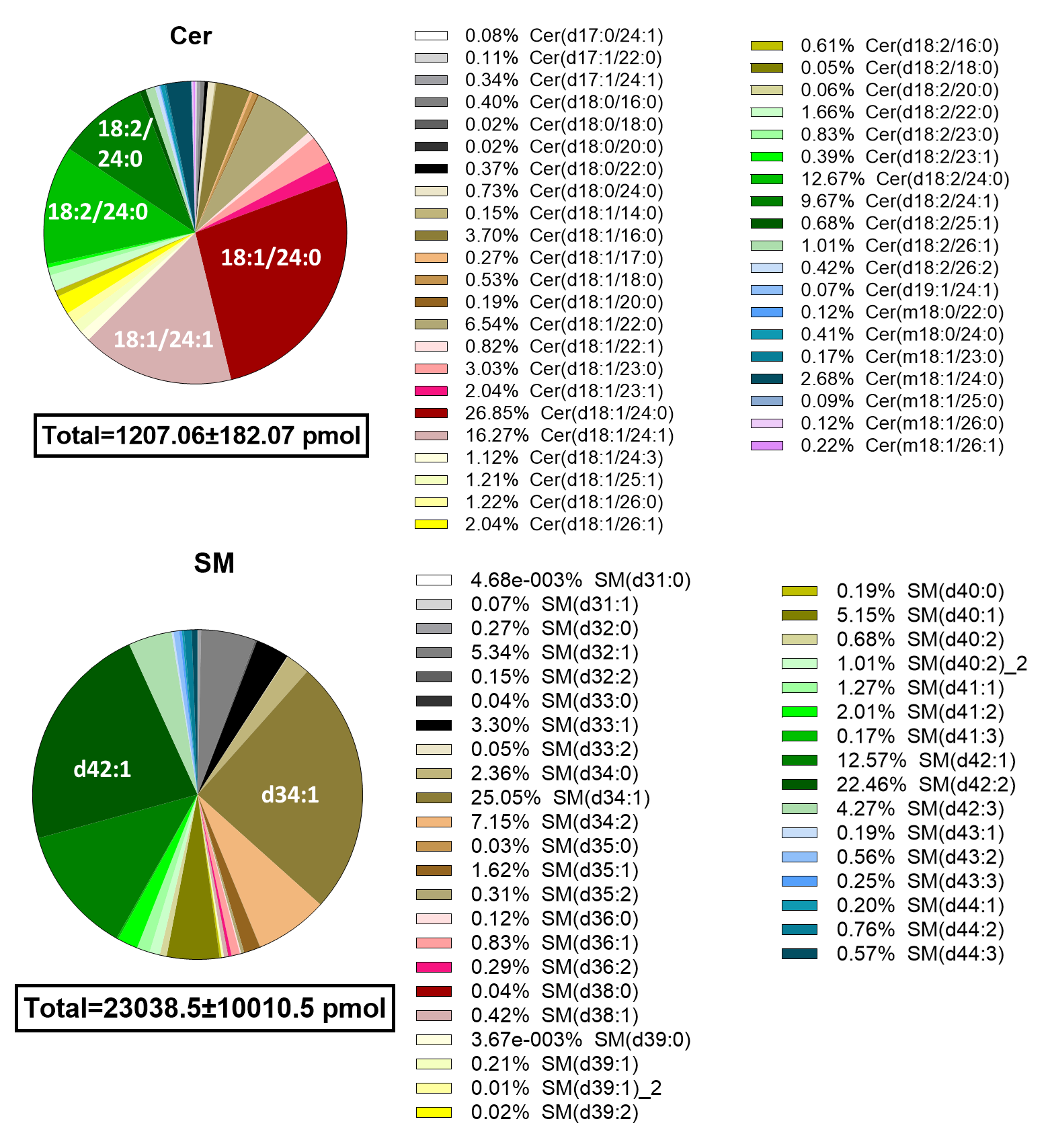


**Figure S13.** Ceramides and sphingomyelins distribution in control fibroblasts. Total amount is shown in control fibroblast and represent the pmoles amount per 1 million cells. The total protein conc for 1 million cells was 0.34 mg. The current levels are in agreement with previous published ceramides levels (1- 9 nmoles/mg protein in fibroblast cells) in fibroblast cells (J Lipid Res. 2007 Feb;48(2):417-24.) Note: the concentrations of all SMs were calculated based on SM(d36:2-d9), so the actual level of very long chain SM is underestimated.
